# Rebamipide Reprograms Hepatic Networks to Prevent and Reverse Metabolic-Dysfunction-Associated Steatotic Liver Disease: Multi-Omics Insights and Histological Validation

**DOI:** 10.3390/ph19040559

**Published:** 2026-03-31

**Authors:** Hebatallah H. Abo Nahas, Abdullah Al-Dakhil, Doaa I. Mohamed, Tarek A. Yousef, Ali H. Abu Almaaty, Ibrahium M. El-Deen, Hatem Adel M. Sembawa, Essa M. Saied

**Affiliations:** 1 Zoology Department, Faculty of Science, Port Said University, Port Said 42526, Egypt; heba.hassan@sci.psu.edu.eg (H.H.A.N.); ali_zoology_2010@yahoo.com (A.H.A.A.); 2 Chemistry Department, College of Science, Imam Mohammad Ibn Saud Islamic University (IMSIU), Riyadh 11623, Saudi Arabia; aaaldakhil@imamu.edu.sa (A.A.-D.); tayousef@imamu.edu.sa (T.A.Y.); 3 Department of Clinical Pharmacology and Therapeutics, Faculty of Medicine, Ain Shams University, Cairo 11566, Egypt; doaapharma@med.asu.edu.eg; 4 Chemistry Department, Faculty of Science, Port Said University, Port Said 42526, Egypt; ieldeen@yahoo.com; 5 Department of General Surgery, Faculty of Medicine, Umm Al-Qura University, Makkah 24382, Saudi Arabia; hasembawa@uqu.edu.sa; 6 Chemistry Department, Faculty of Science, Suez Canal University, Ismailia 41522, Egypt; 7 Institute for Chemistry, Humboldt Universität zu Berlin, 12489 Berlin, Germany

**Keywords:** Rebamipide, MASLD, liver proteomics, lipid metabolism, mitochondrial stress response, metabolic reprogramming

## Abstract

**Background:** Metabolic-dysfunction-associated steatotic liver disease (MASLD) is a growing global health burden, yet no approved pharmacological therapy currently exists. Purpose: The purpose of this study is to investigate the prophylactic and therapeutic potential of Rebamipide, a mucosal-protective and anti-inflammatory drug, in a high-fat diet (MHFD)-induced MASLD rat model, integrating quantitative liver proteomics, network analysis, and histopathology. **Methods:** Male Wistar rats were fed MHFD for 16 weeks and treated with Rebamipide either prophylactically (Reb T1, co-administered with diet) or therapeutically (Reb T2, administered post-NASH onset). Label-free LC-MS/MS proteomics combined with principal component analysis (PCA), partial squares discriminant analysis (PLS-DA), and enrichment analyses (including Gene Ontology (GO), Kyoto Encyclopedia of Genes and Genomes (KEGG), Reactome via g: Profiler, network mapping, and Rat Genome Database (RGD) mining) revealed that MHFD had the following impacts: it induced the profound suppression of mitochondrial chaperones (Hspa9), microsomal triglyceride transfer protein (Mttp), and cytochrome P450 isoforms (Cyp2c6); it disrupted lipid trafficking, oxidative stress defense, and xenobiotic metabolism. **Results:** Rebamipide prophylaxis preserved lipid-handling proteins, prevented glycogen loss, and maintained antioxidant defenses. In contrast, therapeutic administration reversed established steatosis and remodeled metabolic pathways, enhancing fatty acid β-oxidation, detoxification, and mitochondrial protein import. Nine shared proteins across all comparisons, including MTTP and multiple Stress-70 mitochondrial isoforms, mapped to three core genes (Mttp, Cyp2c6, Hspa9) central to lipid transport, protein import, and metabolic stress adaptation. KEGG and Reactome analyses highlighted Rebamipide’s modulation of bile acid synthesis, ceramide and phosphatidylcholine metabolism, lipoprotein remodeling, and MAPK signaling. Histopathological evaluation confirmed Rebamipide’s efficacy, showing reduced steatosis and the normalization of the hepatocyte structure, with near-complete restoration in the therapeutic (Reb T2) group compared to partial protection in the Reb T1 group. **Conclusions:** These findings demonstrate Rebamipide’s dual-phase, multi-targeted mechanism: early protection against diet-induced metabolic injury and robust reversal of established MASLD pathology. The identified protein triad (Mttp, Cyp2c6, Hspa9) and associated pathways provide novel biomarker candidates and mechanistic insight supporting Rebamipide’s repurposing as a therapeutic for metabolic liver disease.

## 1. Introduction

Metabolic-dysfunction-associated steatotic liver disease (MASLD), formerly termed non-alcoholic fatty liver disease (NAFLD), represents the hepatic manifestation of metabolic syndrome and is closely linked to obesity, insulin resistance, systemic inflammation, and increased cardiovascular risk, including advanced atherosclerosis [1]. The global prevalence of MASLD is rising rapidly, affecting over a quarter of the adult population and emerging as the leading cause of chronic liver disease worldwide. The pathogenesis of MASLD remains incompletely understood but is widely explained by the “multiple hits” hypothesis, which proposes that hepatic lipid accumulation triggers steatosis, sensitizing the liver to further insults, including adipokine dysregulation, oxidative stress, lipotoxicity, and impaired glucose and lipid metabolism, ultimately leading to non-alcoholic steatohepatitis (NASH), fibrosis, cirrhosis, and hepatocellular carcinoma [2]. In 2020, the diagnostic criteria were updated under the term MASLD, emphasizing the central role of metabolic dysfunction rather than the exclusion of other liver disease etiologies [3]. Early diagnosis and accurate risk stratification are critical for identifying patients most likely to progress to advanced liver disease, yet barriers persist, including variability in diagnostic criteria, limited access to specialized care, and the high cost of emerging therapies. Ongoing research is focused on developing noninvasive biomarkers, imaging techniques, and targeted treatments to address this unmet need [4]. In this context, proteomics has become a powerful tool for elucidating MASLD pathogenesis, enabling the discovery of disease-specific biomarkers, clarifying mechanisms of disease progression, and identifying systemic metabolic alterations that extend beyond the liver [5]. Recent studies have revealed distinct proteomic signatures associated with MASLD progression, fibrogenesis, and metabolic dysfunction, providing a molecular framework for precision diagnostics and therapy development. In liver proteomic profiling, MASLD is characterized by increased expression of proteins involved in immune activation (e.g., complement system components) and extracellular matrix (ECM) remodeling driven by hepatic stellate and endothelial cells, coupled with downregulation of hepatocyte-driven metabolic pathways, including lipid and glucose metabolism [6,7]. These findings highlight the complex interplay of metabolic, inflammatory, and fibrogenic networks in MASLD and underscore the urgent need for multi-pathway therapeutic approaches.

MASLD management remains challenging, as current strategies rely primarily on lifestyle modifications, such as sustained weight loss, dietary adjustments, and physical activity, which are difficult to maintain [8,9]. Although several pharmacological candidates, including insulin sensitizers, antioxidant agents, and antifibrotic therapies, are under investigation, no globally approved drug therapy for NASH currently exists. MASLD progression is increasingly linked to gut–liver axis dysfunction, where gut dysbiosis, increased intestinal permeability, and translocation of bacterial metabolites exacerbate hepatic inflammation and metabolic stress [10]. Rebamipide, a 2(1H)-quinolinone derivative originally developed as a gastroprotective agent, has a well-established safety profile and multiple pleiotropic mechanisms that make it a compelling candidate for repurposing in metabolic liver disease. It enhances gastric mucosal defense by stimulating mucus glycoprotein secretion, promoting epithelial cell proliferation and migration, and accelerating mucosal repair [11,12]. Beyond mucosal protection, Rebamipide exhibits potent antioxidant activity, directly scavenging reactive oxygen species and activating the nuclear factor erythroid 2-related factor 2 (NRF2) pathway. Simultaneously, it suppresses proinflammatory signaling through the nuclear factor kappa light-chain enhancer of activated B cells (NF-κB) inhibition and reduces cytokines, such as interleukin-8 [11,12]. Additionally, Rebamipide modulates AMP-activated protein kinase (AMPK) signaling, increases cyclooxygenase-2 (COX-2) expression, and promotes prostaglandin synthesis, further supporting tissue resilience and repair [13,14]. Recent studies have expanded its therapeutic scope beyond gastrointestinal disease, demonstrating that Rebamipide modulates gut microbiota composition, restores microbial diversity, protects epithelial barrier integrity, and reduces NSAID-induced enteropathy [15]. In experimental MASLD models, Rebamipide administration reduced serum alanine aminotransferase (ALT) and aspartate aminotransferase (AST)levels, alleviated hepatic steatosis, and improved systemic lipid profiles, suggesting beneficial effects on both metabolic regulation and liver injury [16]. These antioxidant, anti-inflammatory, and barrier-protective actions indicate that Rebamipide could address several key drivers of MASLD pathogenesis. However, its precise molecular mechanisms in the liver remain undefined, and there is currently no clinical or regulatory evidence supporting its use in MASLD, highlighting the need for comprehensive mechanistic studies.

Given the multifactorial nature of MASLD and the lack of approved pharmacotherapies, there is a critical need for effective, accessible, and multi-targeted therapeutic strategies. Based on these facts and our interest in drug discovery research [17,18,19,20,21,22], this study aimed to investigate the prophylactic and therapeutic potential of Rebamipide in a nutritionally induced MASLD model. Toward this, we applied an integrative approach combining quantitative liver proteomics, gene network analysis, pathway enrichment, and histopathology to characterize both therapeutic (Reb T1) and prophylactic (Reb T2) interventions. By mapping global proteomic changes in response to a model high-fat diet (MHFD) and subsequent Rebamipide treatment, we identified molecular pathways linked to lipid metabolism, oxidative stress, and inflammatory regulation. This systems-level investigation provides mechanistic insight into Rebamipide’s action in MASLD, defines candidate biomarkers of therapeutic response, and highlights its potential as a repurposable therapy for metabolic liver disease.

## 2. Results

### 2.1. Quality Control and Data Processing

Quality control (QC) and preprocessing of proteomic data were performed for all experimental groups (Supplementary Materials, Figure S1). Proteins with missing values (NAs) in ≥2 samples were excluded, and features with >50% missing values per group were removed from further analysis. The remaining features were subjected to median random imputation within ±1% of the group median. To improve interpretability and visualization, auto-scaling was applied. As shown in the Supplementary Materials, Figure S1A–C, the data distribution across groups (control vs. MHFD, MHFD vs. Reb T1, and MHFD vs. Reb T2) was visualized before and after preprocessing, confirming the improved uniformity and reliability of the dataset.

### 2.2. Exploratory Analysis of Experimental Groups

**Figure 1:**
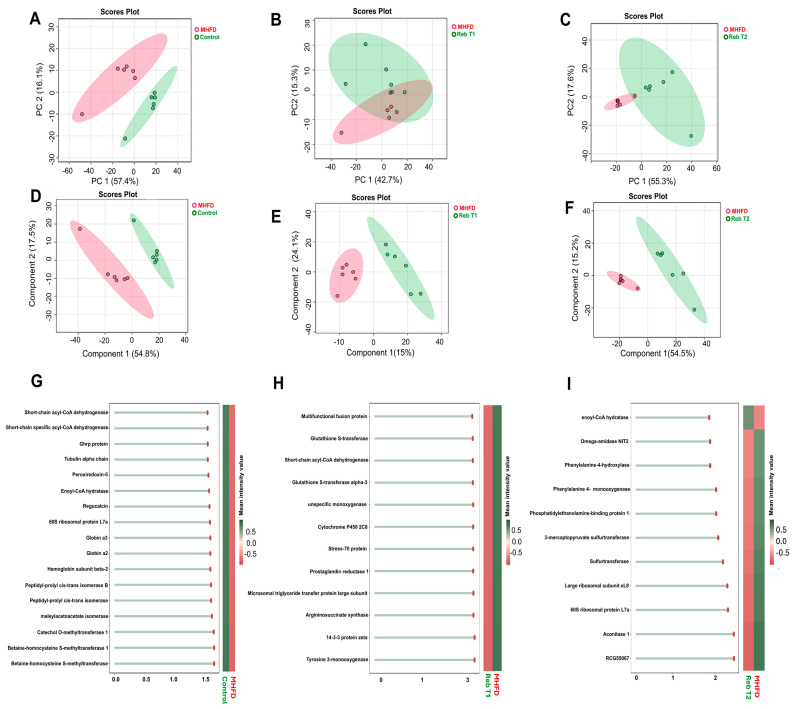
Exploratory analysis of proteomic profiles of MHFD versus control, prophylactic group (Reb T1), and therapeutic group (Reb T2). (**A**–**C**) Principal component analysis (PCA) showing unsupervised distribution of MHFD and control (**A**), MHFD and Reb T1 (**B**), and MHFD and Reb T2 (**C**) groups. (**D**–**F**) Partial least squares discriminant analysis (PLS-DA) 2D score plot of components 1 and 2, illustrating separation between MHFD and control (**D**), MHFD and Reb T1 (**E**), and MHFD and Reb T2 (**F**) groups. (**G**–**I**) Variable importance in projection (VIP) scores (>1.5) derived from PLS-DA, highlighting proteins that contributed most to a MHFD and control (**G**), MHFD and Reb T1 (**H**), and MHFD and Reb T2 (**I**) groups discrimination.

**Figure 2:**
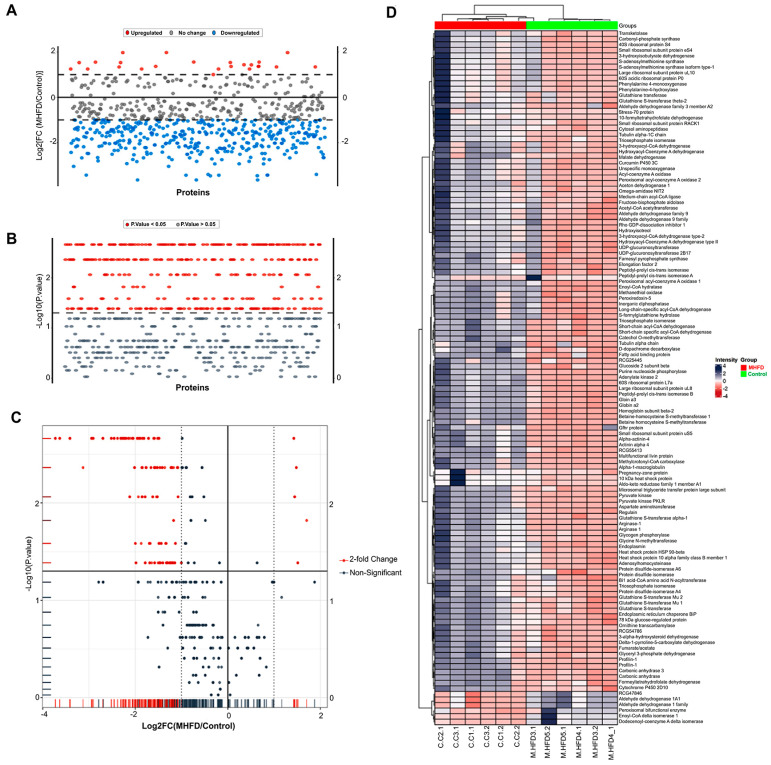
Differential proteomic analysis of control vs. MHFD model. (**A**) Fold-change distribution of proteins. (**B**) Statistical significance determined by the Mann–Whitney test with FDR correction. (**C**) Volcano plot of proteins based on fold change and adjusted significance. (**D**) Heatmap of significantly altered proteins illustrating clustering of control and MHFD samples.

To evaluate both the prophylactic potential of Rebamipide, administered concurrently with HFD, and its therapeutic efficacy when given after NASH development, exploratory multivariate analyses were performed. The aim was to visualize global proteomic changes and determine whether Rebamipide intervention, either preventive or therapeutic, modulates the MHFD-induced hepatic signature. Principal component analysis (PCA) revealed clear separation between the MHFD and control groups, indicating a distinct proteomic profile associated with metabolic stress (Figure 1). This separation was further accentuated by partial least squares discriminant analysis (PLS-DA), which identified high VIP-score proteins such as short-chain acyl-CoA dehydrogenase, enoyl-CoA hydratase, and betaine–homocysteine S-methyltransferase. These discriminatory proteins highlight disrupted β-oxidation, heme metabolism, and one-carbon cycling in MHFD-fed rats. Prophylactic Rebamipide treatment (Reb T1, 100 mg/kg administered concurrently with HFD) partially protected the hepatic proteome from MHFD-induced disruption (Figure 2). Unsupervised PCA demonstrated moderate overlap between the Reb T1 and control groups, indicating that prophylaxis preserved elements of the control proteomic profile but did not achieve full protection. In contrast, supervised PLS-DA more clearly distinguished the groups while highlighting variable importance in projection (VIP) markers linked to oxidative stress defense and lipid metabolism that were maintained or partially normalized under Reb T1. These included the multifunctional fusion protein, glutathione S-transferases, cytochrome P450 family members, and Stress-70 protein. The preservation of these proteins suggests that Rebamipide prophylaxis can attenuate MHFD-induced oxidative stress and maintain lipid-handling capacity, thereby protecting hepatic homeostasis during disease initiation. This indicates that Rebamipide, when given concurrently with dietary challenge, has significant preventive efficacy in mitigating MASLD progression. On the other hand, therapeutic Rebamipide treatment (Reb T2, 100 mg/kg administered after NASH development) produced a more pronounced shift in the hepatic proteome compared to prophylactic treatment (Figure 2). PCA demonstrated complete separation between the Reb T2 and MHFD groups, indicating a strong reversal of MHFD-induced proteomic alterations. Supervised PLS-DA identified a distinct set of high-impact proteins with elevated VIP scores, including enoyl-CoA hydratase, omega-amidase (NIT2), phenylalanine-4-hydroxylase, phosphatidylethanolamine-binding protein 1, and 3-mercaptopyruvate sulfurtransferase. These proteins regulate lipid catabolism, amino acid metabolism, and redox balance, suggesting that therapeutic Rebamipide drives broad metabolic reprogramming to counteract MHFD-driven stress. Collectively, the multivariate analyses demonstrate that MHFD induces substantial disruption of the hepatic proteome. While prophylactic Rebamipide treatment (Reb T1) provides only partial protection, primarily by maintaining antioxidant and lipid-handling pathways, therapeutic administration (Reb T2) achieves more extensive remodeling of metabolic networks, reversing many of the maladaptive changes associated with established disease. These findings highlight Rebamipide’s therapeutic capacity to restore hepatic metabolic integrity and ameliorate molecular dysfunction in MASLD.

### 2.3. Comparative Proteomics Between Control and MHFD Groups to Identify Key Disease Biomarkers

To define the proteomic alterations associated with high-fat-diet-induced hepatic dysfunction, differential expression analysis was performed between the control and MHFD groups (Figure 2). The results revealed a striking suppression of protein abundance in the MHFD model, with more than 70 proteins significantly downregulated relative to controls. Fold-change values ranged from ~0.075 to 0.27, corresponding to log2FC values between −3.7 and −1.9. Among the most severely affected were arginase and arginase-1, which exhibited an approximate 92.5% reduction in abundance (FC ≈ 0.075, log2FC ≈ −3.73). These enzymes are central to the urea cycle and nitrogen disposal, and their loss suggests profound impairment in ammonia detoxification and nitric oxide homeostasis. Similarly, pyruvate kinase isoforms, including PKLR, showed substantial downregulation (log2FC ≈ −3.41), reflecting a disruption of glycolytic flux and reduced cellular energy generation. The collective pattern indicates that MHFD induces not only lipid accumulation but also a systemic metabolic shutdown, characterized by inhibition of nitrogen metabolism, reduced glycolysis, and broad enzymatic suppression. These proteomic changes are in agreement with pathological features reported in MASLD/NASH patients, including impaired nitrogen handling, glycolytic inflexibility, and mitochondrial dysfunction, underscoring the translational relevance of this model.

**Figure 3:**
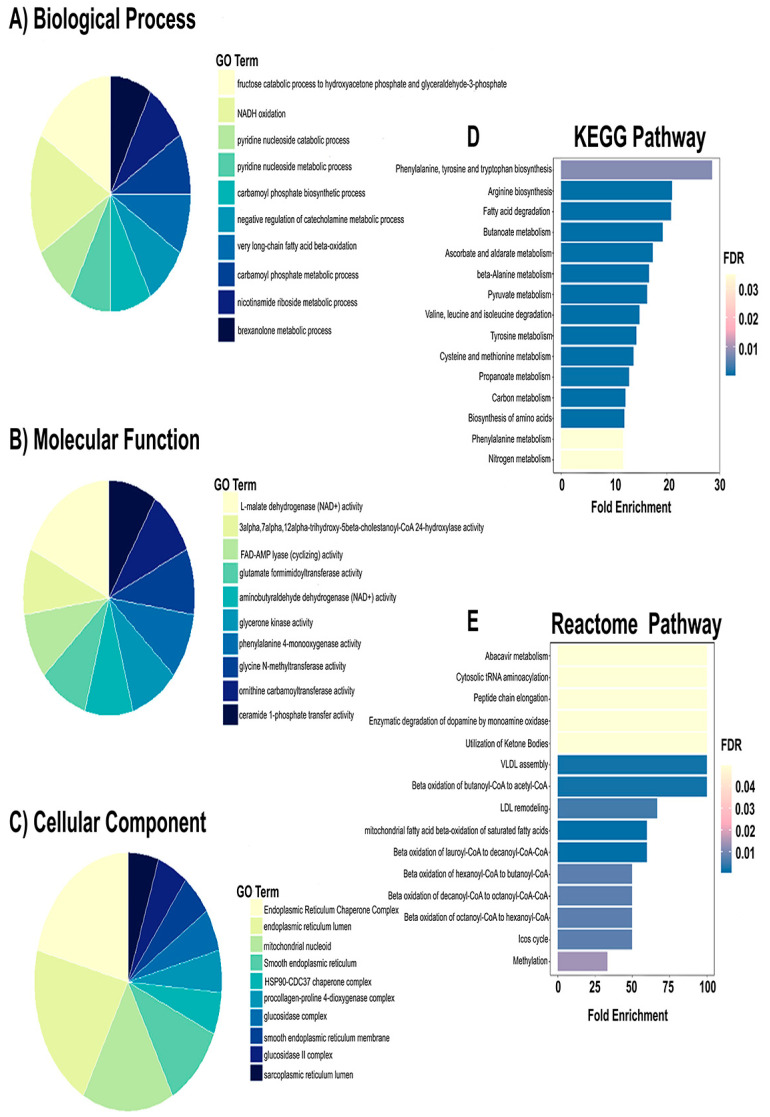
Gene Ontology (GO) and functional enrichment analysis of significantly altered proteins in control versus MHFD groups. (**A**–**C**) GO categories for biological process (BP), molecular function (MF), and cellular component (CC). (**D**,**E**) Pathway enrichment results for KEGG and Reactome.

To further interpret the proteomic alterations between the control and MHFD groups, GO enrichment analysis was performed to identify over-represented biological processes, molecular functions, and pathways (Figure 3). Results showed a pronounced and significant enrichment of metabolic processes, particularly those related to small molecule processing and carboxylic/organic acid metabolism. The biological process term with the largest enrichment factor was carboxylic acid metabolic process, with 34 overlapping proteins out of 81 tested. Closely related terms, including oxoacid metabolic process and organic acid metabolic process, were also highly enriched. Additional enriched categories, such as small molecule catabolic process, organic acid catabolic process, cellular catabolic process, and amino acid metabolic process, indicated a broader reprogramming of nutrient breakdown and degradation in the MHFD condition. At the molecular function level, catalytic activity was significantly enriched with 63 intersecting proteins, reflecting an upregulation of enzyme-mediated biochemical activity. KEGG pathway analysis revealed that the most enriched pathway was fatty acid degradation (FDR = 3.66 × 10^−10^, fold enrichment = 20.83), together with aromatic amino acid biosynthesis, highlighting coordinated disruption of both lipid and amino acid metabolism. Reactome pathway mapping further demonstrated >100-fold enrichment in processes such as β-oxidation of butanoyl-CoA, VLDL assembly, and ketone body utilization, pointing to enhanced lipid catabolism and altered energy metabolism. Notably, enrichment of VLDL assembly implicates MHFD in the remodeling of lipid and lipoprotein formation. Collectively, these enrichment results reveal that MHFD imposes a metabolic reprogramming signature dominated by alterations in organic acid turnover, fatty acid degradation, and lipoprotein assembly. This highlights a pathological shift toward excessive nutrient catabolism and dysregulated lipid handling, features strongly aligned with the metabolic dysfunction observed in MASLD.

### 2.4. Comparative Proteomics of Rebamipide Intervention in MHFD: Prophylactic Versus Therapeutic Effects

#### 2.4.1. Effect of Prophylactic Rebamipide Treatment

**Figure 4:**
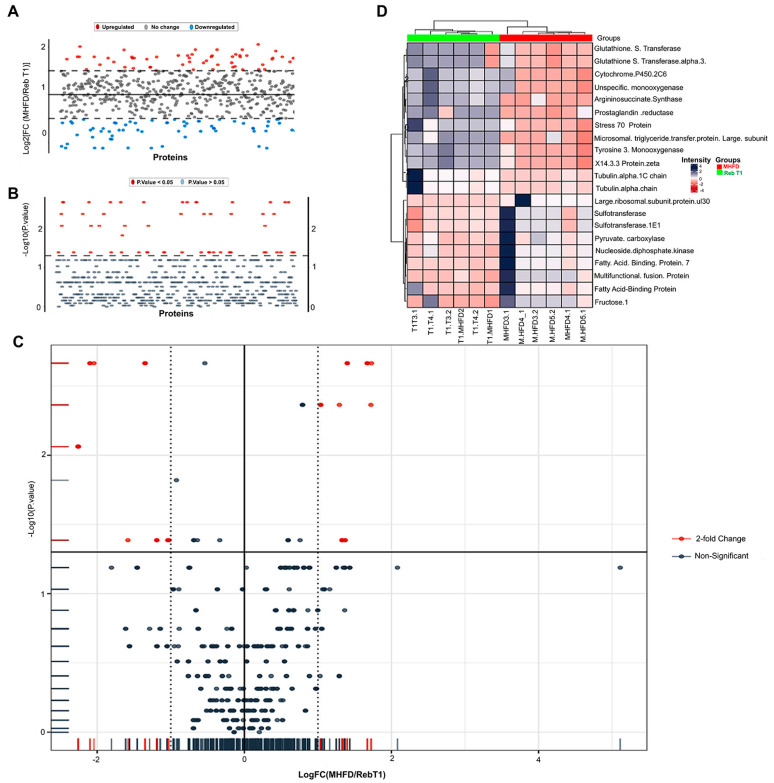
Differential proteomic analysis of MHFD versus prophylactic Rebamipide treatment (Reb T1). (**A**) Fold-change distribution of proteins. (**B**) Statistical significance determined the Mann–Whitney test with FDR correction. (**C**) Volcano plot showing proteins based on fold change and adjusted significance. (**D**) Heatmap of significantly altered proteins illustrating sample clustering of MHFD and Reb T1 groups.

To evaluate the preventive potential of Rebamipide against MHFD-induced proteomic disturbances, we compared the MHFD group with the prophylactic group (Reb T1, 100 mg/kg administered concurrently with HFD). Comparative proteomic analysis revealed distinct changes in protein expression profiles, reflecting both the metabolic stress imposed by MHFD and the protective effects of Rebamipide prophylaxis (Figure 4). A striking feature was the strong downregulation of fatty acid binding protein 7 (FABP7, brain isoform CRA_b) and multifunctional fusion protein in the MHFD group (log_2_FC = −2.10 to −2.04, *p* = 0.002). FABP7 acts as a chaperone that facilitates intracellular trafficking of long-chain fatty acids, and its loss implies reduced fatty acid uptake and lipid handling. Rebamipide prophylaxis appeared to maintain their expression closer to control levels, suggesting preservation of hepatic lipid homeostasis. Conversely, proteins associated with metabolic stress and fibrogenic signaling, including argininosuccinate synthase, tubulin alpha-1C chain, and tubulin alpha chain, were significantly upregulated in MHFD (log_2_FC = 1.67–1.73), consistent with ongoing MASLD progression. Rebamipide prophylaxis normalized their abundance, indicating reduced metabolic burden and protection of hepatocellular resilience. Moderate but notable changes were also detected in other regulatory proteins. The large ribosomal subunit protein uL30 and related proteins (B0K023/B0K031) were downregulated (log_2_FC ≈ −1.35), reflecting translational suppression, while oxidative stress–responsive proteins, including tyrosine 3-monooxygenase/tryptophan 5-monooxygenase activation protein zeta and 14-3-3 protein zeta/delta were upregulated (log_2_FC ≈ 1.40), reflecting stress adaptation. In addition, pyruvate carboxylase, a key enzyme in gluconeogenesis and lipogenesis, was robustly downregulated, while prostaglandin reductase 1, an inflammatory mediator, was upregulated in the MHFD group, both of which were modulated by Rebamipide prophylaxis. Together, these findings indicate that prophylactic Rebamipide administration partially protects against MHFD-induced proteomic alterations by maintaining lipid-handling proteins, reducing stress- and fibrosis-related markers, and modulating inflammatory mediators. These results highlight the preventive capacity of Rebamipide in early disease stages, supporting its potential to delay the progression of MASLD toward steatohepatitis (MASH) and fibrosis.

**Figure 5:**
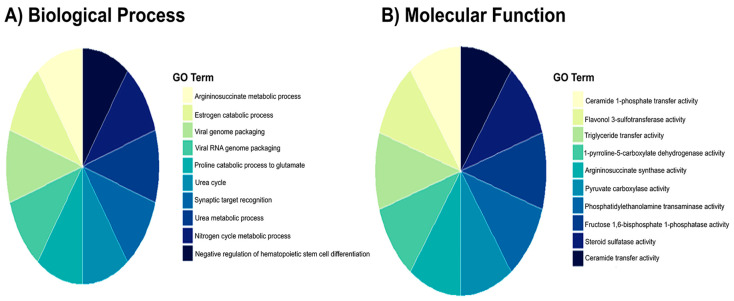
Gene Ontology (GO) enrichment of significantly altered proteins in MHFD versus prophylactic Rebamipide treatment (Reb T1). (**A**) Biological process (BP) categories. (**B**) Molecular function (MF) categories.

**Figure 6:**
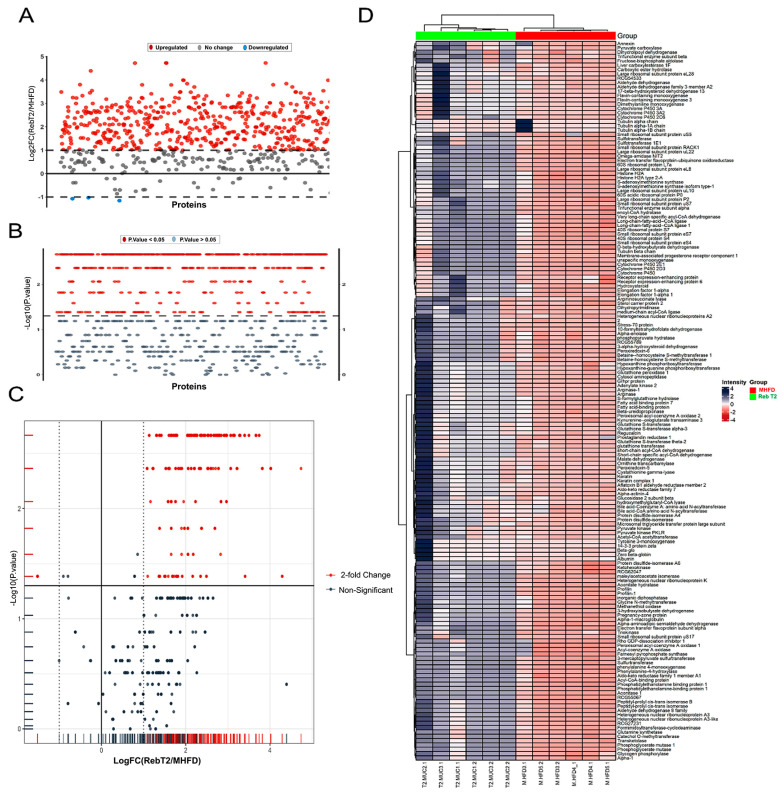
Differential proteomic analysis of MHFD versus therapeutic Rebamipide treatment (Reb T2). (**A**) Fold-change distribution of proteins. (**B**) Statistical significance determined by the Mann–Whitney test with FDR correction. (**C**) Volcano plot of proteins based on fold change and adjusted significance. (**D**) Heatmap of significantly altered proteins illustrating clustering of MHFD and Reb T2 samples.

To explore the functional implications of proteomic changes induced by prophylactic Rebamipide administration, GO enrichment analysis was performed between the MHFD and Reb T1 groups. This analysis is informative because it highlights the biological processes and molecular functions that are protected or modulated when Rebamipide is given concurrently with HFD, thereby revealing pathways that may underlie its preventive action (Figure 5). The results indicated significant enrichment of several biological processes (BP), particularly those linked to organic acid metabolism. The top enriched terms included organic acid metabolic process and oxoacid metabolic process (FDR = 0.001), represented by five intersecting proteins. Additional enrichment was observed for carboxylic acid metabolic process, gluconeogenesis, and purine nucleotide metabolic process (FDR < 0.014), suggesting that Rebamipide prophylaxis helps maintain energy metabolism and nucleotide turnover under MHFD stress. For molecular function (MF), multiple categories showed robust enrichment, with the strongest effects observed in nucleotide binding, purine ribonucleotide binding, and carbohydrate derivative binding (FDR ≈ 0.0066), each represented by six intersecting proteins (Figure 6). Notably, several unique enzyme activities, ceramide 1-phosphate transfer activity, flavonol 3-sulfotransferase activity, and argininosuccinate synthase activity, were extraordinarily enriched, indicating highly specific molecular adaptations. Taken together, these enrichment results suggest that prophylactic Rebamipide treatment influences both broad metabolic processes and specialized enzyme activities. The pattern points to preventive metabolic reprogramming, particularly in organic acid turnover, nucleotide metabolism, and lipid signaling, providing a mechanistic basis for Rebamipide’s protective effects against MHFD-induced hepatic dysfunction.

#### 2.4.2. Effect of Therapeutic Rebamipide Treatment

To evaluate the therapeutic potential of Rebamipide in reversing established MHFD-induced proteomic disturbances, we compared the MHFD group with the therapeutic group (Reb T2, 100 mg/kg administered post-NASH development). This analysis is informative as it identifies the molecular pathways that are restored or remodeled when Rebamipide is applied after disease onset (Figure 6). Differential proteomic analysis revealed clear shifts in protein abundance, with several proteins displaying dramatic fold changes and high statistical significance. Among the most strongly upregulated in the MHFD group were alpha-actinin-4 (log_2_FC = 3.74) and adenylate kinase 2, mitochondrial (log_2_FC = 3.67; *p* = 0.002), both of which are implicated in cytoskeletal remodeling, mitochondrial dysfunction, and inflammatory signaling. Additional increases were observed for phosphoglycerate mutase 1 (log_2_FC = 3.50), consistent with disturbed glycolytic and mitochondrial flux. In contrast, significantly downregulated proteins included tubulin alpha-1A and tubulin alpha-1B chains (log_2_FC = −1.52), indicating impaired insulin signaling and cytoskeletal organization. The volcano plot further highlighted distinct outliers such as 10-formyltetrahydrofolate dehydrogenase (log_2_FC = 4.74) and cytochrome P450 2E1 (log_2_FC = 4.02), suggesting their biomarker potential for MHFD-induced injury. Interestingly, Rebamipide therapy appeared to normalize the abundance of these proteins, implying restoration of mitochondrial bioenergetics (via adenylate kinase 2 and phosphoglycerate mutase 1), reduction in oxidative stress (through cytochrome P450 2E1 suppression), improvement of insulin signaling (via tubulin alpha modulation), and attenuation of inflammation and fibrosis (via alpha-actinin-4 correction). Unsupervised clustering in the heatmap further revealed a coordinated upregulation of alpha hydrolases (AOS1-5, H2O1-170) and mitochondrial proteins in the MHFD group, consistent with adaptive responses to oxidative stress and metabolic dysregulation. Rebamipide therapy reversed this pattern, indicating its role in restoring redox balance, mitigating mitochondrial dysfunction, and reducing lipotoxicity. Collectively, these findings demonstrate that therapeutic Rebamipide administration reverses key proteomic derangements induced by MHFD, restoring pathways linked to mitochondrial bioenergetics, oxidative stress defense, insulin signaling, and cytoskeletal stability. These results highlight Rebamipide’s strong potential as a therapeutic intervention for MASLD progression.

**Figure 7:**
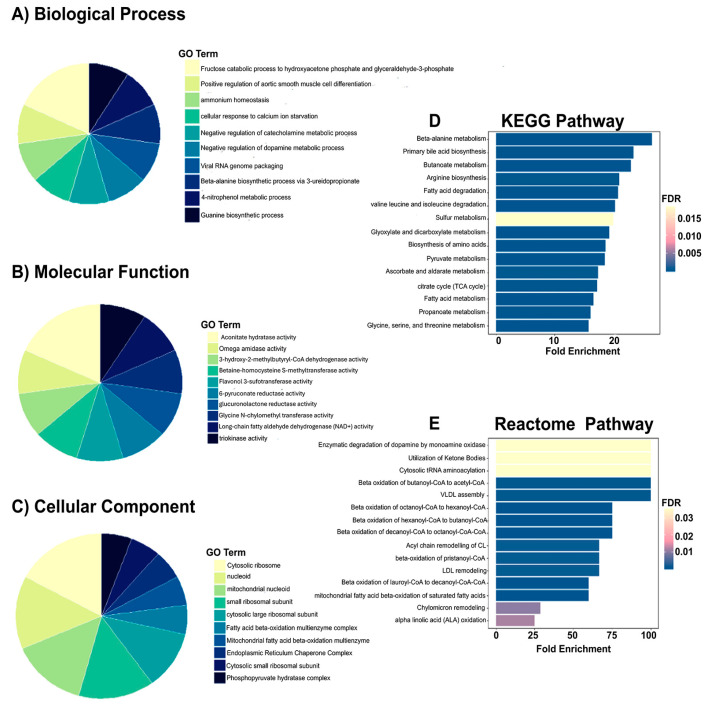
Gene Ontology (GO) and functional enrichment analysis of significantly altered proteins in MHFD versus therapeutic Rebamipide treatment (Reb T2). (**A**–**C**) GO categories for biological process (BP), molecular function (MF), and cellular component (CC). (**D**,**E**) Pathway enrichment results for KEGG and Reactome.

To investigate the functional implications of therapeutic Rebamipide administration, GO enrichment analysis was performed between the MHFD and Reb T2 groups. This analysis is informative as it explores the biological pathways that are restored or remodeled when Rebamipide is administered after NASH development, providing mechanistic insight into its therapeutic actions (Figure 7). At the level of biological processes, significant enrichment was observed in pathways related to organic acid and small molecule metabolism, consistent with metabolic reprogramming. The most enriched term was organic acid metabolic process, followed by small molecule metabolic process (52 proteins) and small molecule catabolic process. These results suggest not only increased degradation of metabolites but also enhanced utilization of metabolic intermediates to maintain cellular homeostasis under MHFD stress. Additional enrichment in monocarboxylic acid metabolism and fatty acid β-oxidation points to increased lipid breakdown and energy generation, while carboxylic acid catabolic process and organic acid catabolic process (each with ~12.9% protein representation) indicate a shift away from biosynthesis toward catabolism, consistent with detoxification and adaptive energy regulation. Molecular function (MF) enrichment highlighted enzyme activities central to therapeutic adaptation. Key enriched terms included 3-hydroxy-2-methylbutyryl-CoA dehydrogenase activity, betaine–homocysteine S-methyltransferase activity, and long-chain fatty aldehyde dehydrogenase (NAD^+^) activity, implicating amino acid metabolism, lipid oxidation, and sulfur compound processing. Additional enriched functions, L-glucuronate reductase activity, glycine N-choloyltransferase activity, and triokinase activity, point to modulation of bile acid conjugation, detoxification pathways, and carbohydrate metabolism. Cellular component (CC) enrichment revealed substantial representation of the fatty acid β-oxidation multienzyme complex and mitochondrial fatty acid β-oxidation multienzyme complex, emphasizing the spatial organization of lipid degradation processes within mitochondria. This suggests a consolidated metabolic response to meet higher energy demands and preserve redox balance. Pathway-level analysis supported these findings. KEGG enrichment identified significant pathways in amino acid and short-chain fatty acid metabolism, including β-alanine metabolism (FDR = 1.5 × 10^−8^, fold enrichment = 26.7), butanoate metabolism, and primary bile acid biosynthesis. Reactome analysis further revealed enrichment in pathways associated with lipid transport and breakdown (VLDL assembly, β-oxidation, ketone body utilization), as well as amino acid biosynthesis (arginine, β-alanine, phenylalanine/tyrosine/tryptophan). These results reveal a broad modulatory role for Rebamipide in supporting hepatic detoxification, lipid handling, and energy balance (Figure 7). Taken together, these enrichment results indicate that therapeutic Rebamipide treatment reprograms central metabolic pathways in MHFD-fed animals, particularly those involved in organic acid turnover, fatty acid oxidation, amino acid metabolism, and bile acid conjugation. This remodeling likely reflects an adaptive therapeutic response that restores hepatic homeostasis and mitigates the molecular dysfunction associated with MASLD.

### 2.5. Proteomic Profiling of Common Protein Abundance Patterns

**Figure 8:**
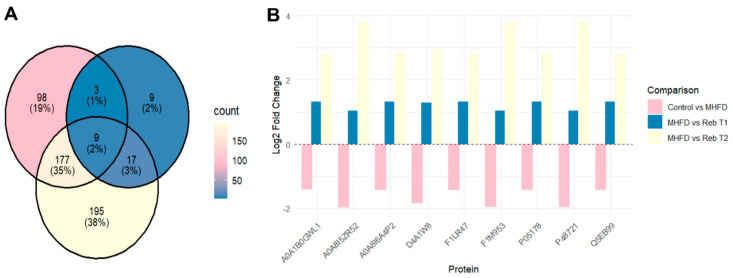
Common proteins modulated by MHFD and Rebamipide treatment. (**A**) Venn diagram of significantly regulated proteins (log_2_FC > 1, FDR < 0.05) across three pairwise comparisons: control vs. MHFD, MHFD vs. prophylactic Rebamipide (Reb T1), and MHFD vs. therapeutic Rebamipide (Reb T2). (**B**) Grouped bar plot of log_2_ fold change for each common protein across the three comparisons, illustrating partial recovery with prophylactic treatment (Reb T1) and stronger reactivation with therapeutic treatment (Reb T2).

To identify robust molecular signatures of MHFD-induced dysfunction and their modulation by Rebamipide, we performed comparative proteomic profiling across all experimental groups (Control, MHFD, prophylactic Reb T1, and therapeutic Reb T2). This analysis is informative as proteins consistently altered across all conditions may represent core disease biomarkers as well as targets of therapeutic actions (Figure 8). Our analysis identified nine common proteins whose expression was consistently dysregulated in the MHFD group and restored following Rebamipide intervention. Four proteins, mainly microsomal triglyceride transfer protein large subunit (D4A1W8) and three isoforms of the mitochondrial stress-70 protein (F1M953, A0A8I5ZR52, and P48721), were significantly downregulated in MHFD (log_2_FC = −1.86 to −1.99). Both prophylactic (Reb T1) and therapeutic (Reb T2) treatment reversed this suppression, with Reb T2 restoring and even overcompensating expression of some stress-70 isoforms (A0A8I5ZR52 and P48721; log_2_FC > +3.8). These findings indicate that while prophylaxis preserves mitochondrial chaperone activity, therapeutic administration can strongly reactivate it in established disease. Further, a second protein cluster included unspecific monooxygenases (F1LR47, Q5EB99, A0A8I6A4P2, A0A1B0GWL1) and cytochrome P450 2C6 (P05178). These were moderately downregulated under MHFD but showed consistent upregulation following Rebamipide treatment, with the most consistent and robust induction observed in the therapeutic group (Reb T2). These proteins are involved in xenobiotic metabolism and lipid processing, suggesting the therapeutic potential of Rebamipide in the restoration of hepatic detoxification capacity impaired by MHFD. Collectively, these findings reveal that MHFD induces marked suppression of proteins involved in lipid transport, mitochondrial proteostasis, and xenobiotic metabolism, while both prophylactic and therapeutic Rebamipide restore their abundance. The similarity of downregulation patterns in MHFD and consistent reactivation by treatment across all nine proteins indicates that they may serve as candidate biomarkers for MHFD-induced dysfunction and for monitoring Rebamipide’s therapeutic or preventive efficacy. The superior restoration achieved with therapeutic Reb T2 demonstrates the potent capacity of Rebamipide to reprogram proteomic alterations once disease is established.

### 2.6. Identification of Candidate Genes Associated with MASLD and Related Metabolic Disorders

**Figure 9:**
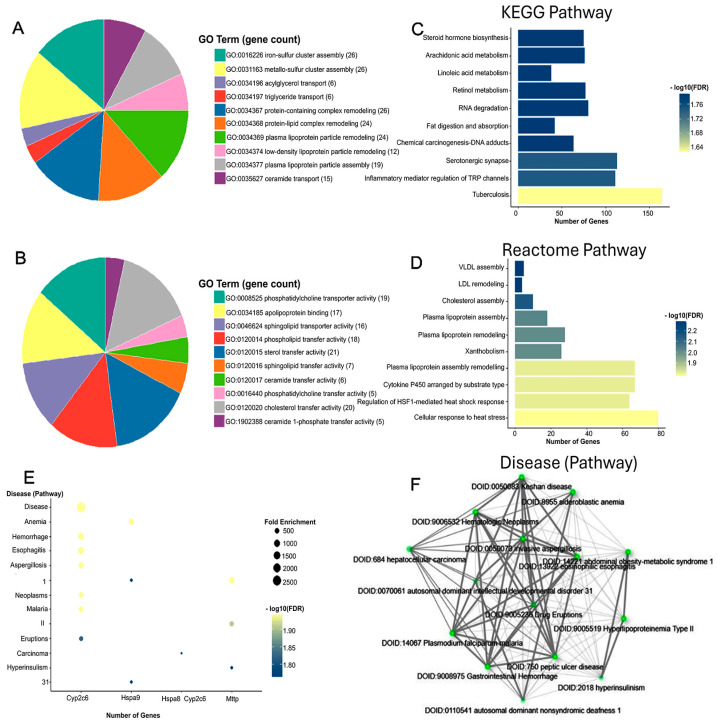
GO and functional enrichment analysis of key regulatory genes. (**A**,**B**) GO categories for biological process (BP) and molecular function (MF). (**C**,**D**) Pathway enrichment analysis for KEGG and Reactome. (**E**) Dot plot summarizing disease annotations. (**F**) Network diagram illustrating the interconnections among enriched disease terms.

To gain deeper insight into the functional relevance of the common proteins identified, we performed data mining in the Rat Genome Database (RGD). This analysis is informative because it links proteomic findings to genetic determinants of metabolic dysfunction, highlighting candidate genes that may contribute to MASLD pathogenesis. As shown in Figure 9, our analysis identified three candidate genes, namely Mttp, Cyp2c6, and Hspa9. The Mttp gene, the rat ortholog of human MTTP, showed strong associations with MASLD, steatotic liver disease in the context of hepatitis C infection, MTTP mice with liver-specific deletion of Mttp (Mttp-LKO) develop hepatic steatosis and complete abrogation of VLDL [23]. Notably, variants such as −493G > T in MTTP have been linked to coronary artery disease and hyperinsulinism, underscoring its systemic role in lipid metabolism and metabolic regulation. The Cyp2c6 gene, corresponding to human CYP2C19, was associated with a range of metabolic and hepatic disorders, including dyslipidemia, hepatocellular carcinoma, and type 2 diabetes. Although no direct association with MASLD has been established, polymorphisms of CYP2C19 have been investigated for their pharmacogenomic relevance and their potential to influence lipid-related pathways, suggesting an indirect contribution to hepatic metabolic dysfunction. The third candidate, Hspa9, encodes a mitochondrial stress protein linked to disorders driven by mitochondrial impairment, including sideroblastic anemia and neurodevelopmental syndromes. While direct evidence for its role in MASLD is lacking, HSPA9’s involvement in mitochondrial protein import and oxidative stress regulation provides a plausible mechanistic link to hepatic steatosis and systemic metabolic imbalance. Collectively, these results reinforce Mttp as a primary MASLD-linked gene, while Cyp2c6 and Hspa9 emerge as secondary candidates. Together, their associations highlight the intersection of lipid metabolism, mitochondrial homeostasis, and xenobiotic pathways as central to MASLD pathophysiology, warranting further validation in metabolic liver disease models.

#### 2.6.1. Network Analysis of Proteomic-Mapped Key Regulatory Genes

**Figure 10:**
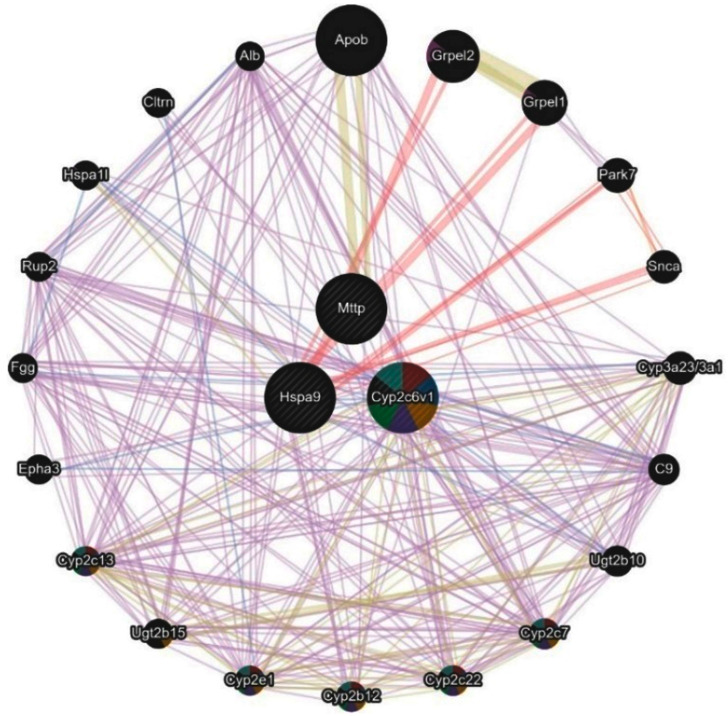
Network analysis of key regulatory genes associated with MHFD and Rebamipide treatment. Interaction network of Mttp, Cyp2c6, and Hspa9 **node** represents an individual gene, highlighting their functional associations. **Edges** are Co-expression links (39.02%) that connect these genes with lipid metabolism (e.g., Apob, Grpel2) and detoxification pathways (e.g., Cyp3a23/3a1, Ugt2b10). Physical interactions (25.61%) associate Hspa9 with mitochondrial import proteins (Grpel1/2) and ubiquitination (TRIM21). Predicted interactions (21.99%) link the genes to innate immunity and MAPK signaling, supporting their role in oxidative stress adaptation and inflammation resolution.

To further explore the functional relevance of the candidate genes identified, network analysis was performed for Mttp, Cyp2c6, and Hspa9. This analysis is informative as it highlights the interaction partners and biological processes through which these genes may contribute to MHFD-induced pathology and Rebamipide’s protective effects. As shown in Figure 10, the network revealed strong contributions from co-expression interactions (39.02%), linking these genes to lipid metabolism (Apob, Grpel2) and detoxification pathways (Cyp3a23/3a1, Ugt2b10). These associations suggest that Rebamipide may reverse MHFD-induced transcriptional repression of lipid-handling and detoxification machinery. Physical interactions (25.61%) further connected Hspa9 with mitochondrial protein import (Grpel1/2) and ubiquitination processes (TRIM21), implying that Rebamipide stabilizes mitochondrial integrity and proteostasis. Additionally, predicted interactions (21.99%) linked these genes to innate immunity and MAPK signaling, consistent with a role in resolving inflammatory responses. Together, these interaction modes suggest that Rebamipide modulates detoxification, mitochondrial import, and stress-adaptive pathways to attenuate oxidative stress and maintain hepatic homeostasis. Interestingly, the analysis also suggested treatment-specific mechanisms. Prophylactic Rebamipide (Reb T1) may rapidly activate detoxification through Cyp2c6 and stress-response pathways via Hspa9, thereby buffering the liver against early MHFD-induced insults. In contrast, therapeutic Rebamipide (Reb T2) appears to more strongly restore Mttp-mediated lipid trafficking, contributing to the resolution of steatosis and metabolic dysfunction in established disease. Collectively, the network analysis implicates Cyp2c6, Hspa9, and Mttp as central regulators of MHFD-induced metabolic dysregulation and as key molecular targets through which Rebamipide exerts both its preventive and therapeutic actions.

#### 2.6.2. Enrichment Analysis of Shared Proteomic Genes in Disease and Rebamipide Treatment Conditions

To further characterize the biological significance of the key regulatory genes and proteins, enrichment analysis was performed across disease and treatment conditions. This analysis is informative because it highlights the metabolic and pathological pathways most strongly affected by MHFD and modulated by Rebamipide, thereby providing a mechanistic context for their role in MASLD. As shown in Figure 9, the GO biological process (BP) enrichment revealed strong associations with lipid metabolism, including triglyceride transport, lipoprotein localization, ceramide metabolism, and protein–lipid complex assembly (Figure 9A). These processes reflect the lipid-handling defects and metabolic stress characteristic of MHFD-induced liver disease. GO molecular function (MF) enrichment pointed to activities related to lipid transfer, such as phosphatidylcholine, ceramide, and cholesterol transport, along with chaperone activity involved in protein folding (Figure 9B). These findings indicate both lipid dysregulation and activation of stress-adaptive mechanisms, including ER stress responses that contribute to inflammation and steatosis. At the pathway level, KEGG and Reactome analyses identified enrichment in fatty acid metabolism (notably linoleic and arachidonic acid metabolism), fat digestion and absorption, steroid hormone biosynthesis, and lipoprotein remodeling (Figure 9C,D). Pathways related to xenobiotic metabolism, oxidative stress defense, and inflammatory signaling were also enriched, consistent with the combined metabolic and immune perturbations driving MASLD progression. Finally, disease enrichment analysis highlighted links to abdominal obesity, metabolic syndrome, hyperlipoproteinemia type II, hyperinsulinism, and hepatocellular carcinoma (Figure 9E,F). These associations underscore the translational relevance of MHFD-induced models and reveal how Rebamipide may intervene in pathways connecting metabolic dysfunction to liver disease and malignancy. Collectively, these enrichment results demonstrate that the shared proteomic genes are central to lipid metabolism, stress adaptation, and immune signaling. Their dysregulation under MHFD and partial restoration by Rebamipide reinforce their role as key molecular nodes bridging metabolic stress and MASLD progression.

### 2.7. Histopathological Evaluation of Liver Tissues

**Figure 11:**
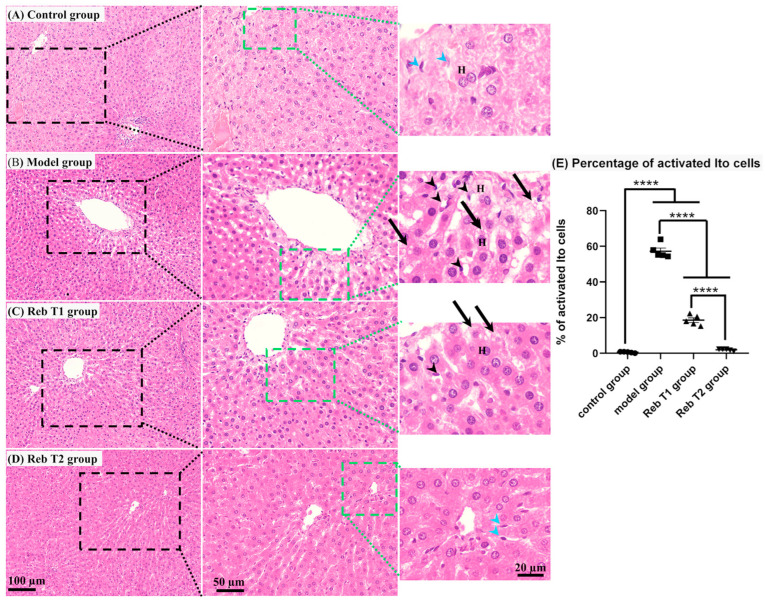
Histopathological and morphometrical analysis of rat liver sections stained with H&E. Representative photomicrographs from Control (**A**), MHFD (Model) (**B**), Reb T1 (prophylactic) (**C**), and Reb T2 (therapeutic) (**D**) groups. The Control group (**A**) shows normal hepatic architecture with a central vein (CV), radiating hepatocyte plates (H), and quiescent stellate (Ito) cells (blue arrowheads). The MHFD Model (**B**) and Reb T1 (**C**) groups display hepatocytes with cytoplasmic fat droplet accumulation (arrows) and activated Ito cells containing lipid vacuoles (black arrowheads). The Reb T2 group (**D**) demonstrates restoration of near-normal hepatic architecture with reduced steatosis and stellate cell activation. (**E**) Graph showing the morphometrical data of the percentage of activated Ito cells in H&E-stained liver sections of the different studied groups. Highly significant (****) values were detected at *p*-values < 0.01, *n* = 5/experimental group. Statistical analysis was performed using the two-way analysis of variance (ANOVA) followed by Tukey’s post hoc multiple comparison test. Data are presented as mean ± standard error (SEM). Higher-magnification panels represent zoomed views of the boxed regions from the same microscopic field to illustrate detailed hepatocellular morphology.

To validate the proteomic findings and evaluate the structural effects of Rebamipide on MHFD-induced liver injury, histological examination of hepatic tissue was performed using hematoxylin and eosin (H&E) staining (Figure 11). This analysis is informative as it provides direct morphological evidence of steatosis, inflammation, and architectural changes that complement the molecular alterations identified in the proteomic datasets. In the control group, liver sections showed normal hepatic architecture with less distinct lobular demarcation, a central vein surrounded by radiating hepatocyte plates, and well-preserved sinusoids. Hepatocytes appeared polygonal with eosinophilic cytoplasm containing fine basophilic granules and one to two centrally located nuclei with prominent nucleoli. The space of Disse displayed quiescent stellate (Ito) cells, consistent with normal physiological conditions (Figure 11A). In contrast, the MHFD model group demonstrated clear pathological changes. Hepatocytes exhibited pale, faintly eosinophilic cytoplasm with numerous intracellular fat droplets, indicative of macrovesicular steatosis. The space of Disse showed an increased number of activated stellate (Ito) cells with lipid accumulation, reflecting fibrogenic activation. In addition, the portal tracts of several sections revealed focal mononuclear cell infiltration, consistent with the inflammatory progression of MASLD (Figure 11B and Figure S3). The Reb T1 group (prophylactic treatment) showed partial protection. Compared with the MHFD group, hepatocytes displayed fewer fat droplets and fewer activated Ito cells, although steatosis was still evident (Figure 11C). These findings suggest that Rebamipide prophylaxis limited, but did not fully prevent, lipid accumulation and stellate cell activation under MHFD challenge. Remarkably, the Reb T2 group (therapeutic treatment) demonstrated marked histological improvement. Liver sections revealed restoration of near-normal hepatic architecture with clearer hepatocyte morphology, reduced lipid deposition, and normalization of the space of Disse. The reduction in Ito cell activation and disappearance of prominent fat vacuoles indicated reversal of steatosis and fibrogenic signaling (Figure 11D). Collectively, these histological findings confirm that MHFD feeding induces classical MASLD features, including steatosis, stellate cell activation, and inflammation. Interestingly, morphometrical analysis of the percentage of activated Ito cells demonstrated a highly significant increase in both the model and Reb T1 groups compared with the control group, whereas no significant difference was observed between the control and Reb T2 groups. Moreover, the Reb T2 group showed a highly significant reduction in the percentage of activated Ito cells compared with both the model and Reb T1 groups. These findings suggest that Rebamipide treatment attenuates hepatic alterations, providing partial protection under prophylactic administration (Reb T1) and marked restoration under therapeutic intervention (Reb T2). These histological and morphometrical outcomes reinforce the proteomic results, supporting Rebamipide’s dual capacity to limit disease initiation and reverse established hepatic injury.

**Figure 12:**
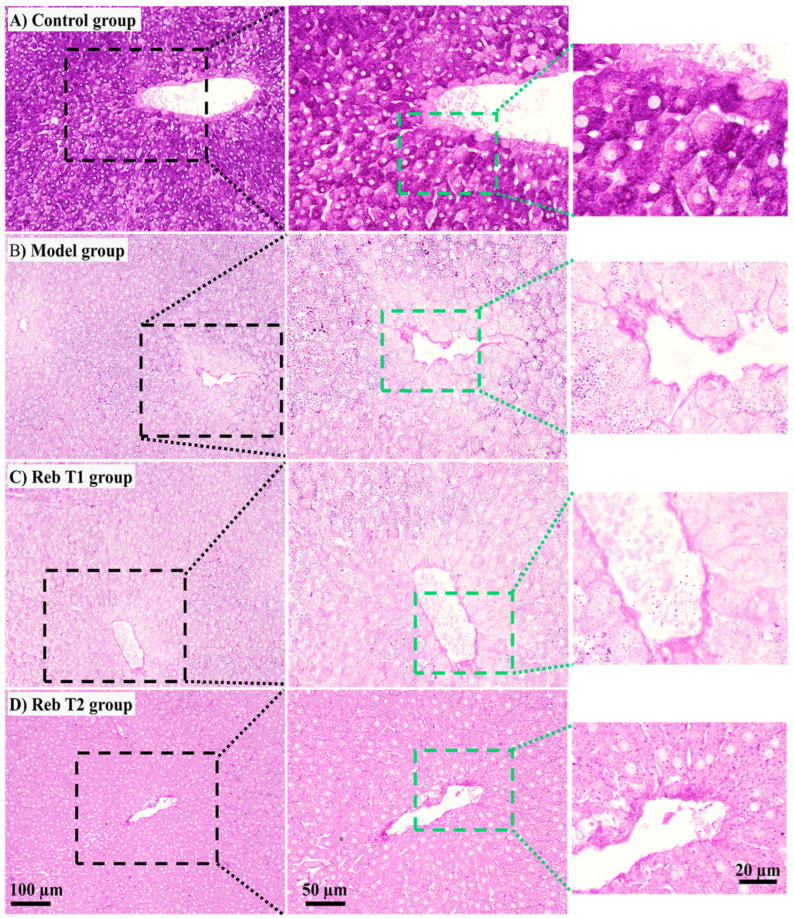
Histopathological analysis of rat liver sections stained with PAS. Representative photomicrographs from Control, MHFD (Model), Reb T1 (prophylactic), and Reb T2 (therapeutic) groups. The Control group shows abundant dark pink glycogen deposits in hepatocyte cytoplasm. In contrast, hepatocytes from the MHFD Model and Reb T1 groups exhibit pale cytoplasm with markedly reduced glycogen deposition, consistent with glycogen depletion and steatotic changes. The Reb T2 group demonstrates restoration of glycogen storage, with hepatocyte cytoplasm displaying intense PAS-positive granules comparable to the Control group. Higher-magnification panels represent zoomed views of the boxed regions from the same microscopic field to illustrate detailed hepatocellular morphology.

To complement H&E staining and further evaluate hepatocellular metabolism, liver tissues were examined using PAS staining to assess glycogen storage. This analysis is informative because glycogen depletion is a hallmark of metabolic dysfunction in MASLD, and restoration of glycogen reserves represents recovery of energy balance and hepatocellular integrity. In the control group, hepatocytes exhibited strong PAS reactivity, with abundant dark pink glycogen granules evenly distributed throughout the cytoplasm. The glycogen deposits appeared diffuse but uniform, giving the hepatocytes a dense, granular appearance. The polygonal cells showed well-preserved cytoplasmic content with clearly defined borders and centrally located nuclei, consistent with healthy metabolic function (Figure 12A). In contrast, hepatocytes from the MHFD model group demonstrated striking glycogen depletion. The cytoplasm appeared pale and faintly stained, with only scattered PAS-positive granules visible. Many hepatocytes showed vacuolated cytoplasm, reflecting fat infiltration replacing normal glycogen stores. The depletion of glycogen coincided with hepatocellular ballooning, loss of cytoplasmic density, and distortion of normal hepatocyte architecture. This pattern is consistent with impaired glucose handling and a shift toward lipid accumulation as an energy source under MHFD challenge (Figure 12B). The Reb T1 group (prophylactic treatment) showed partial preservation of glycogen compared to the MHFD group. While hepatocytes still displayed pale cytoplasm with reduced glycogen deposits, occasional dark pink granules were observed, indicating limited retention of glycogen. However, the overall staining intensity and distribution remained markedly reduced relative to the control. These findings suggest that prophylactic Rebamipide provided some protection against glycogen loss, but was insufficient to fully prevent the metabolic imbalance caused by prolonged MHFD exposure (Figure 12C). Interestingly, the Reb T2 group (therapeutic treatment) exhibited robust restoration of glycogen storage. Hepatocytes displayed intense PAS positivity with abundant dark pink cytoplasmic granules, closely resembling the control group. The staining was more uniform and widespread across hepatocytes, and the cytoplasm appeared denser and more structured, indicating reactivation of glycogen synthesis and storage (Figure 12D). The restoration of glycogen reserves reflects recovery of glucose metabolism and energy homeostasis, likely mediated by the therapeutic action of Rebamipide in reversing MHFD-induced dysfunction. Collectively, the PAS-staining analysis revealed that MHFD feeding depletes hepatocellular glycogen, consistent with impaired glucose metabolism and lipid substitution in MASLD. Prophylactic Rebamipide (Reb T1) provided only limited protection, whereas therapeutic Rebamipide (Reb T2) markedly restored glycogen content, reinforcing its capacity to reverse metabolic imbalance. These morphological observations align with the proteomic and H&E findings, providing strong histological validation of Rebamipide’s role in restoring both lipid and glucose homeostasis in the liver.

## 3. Discussion

The MASLD represents the hepatic manifestation of metabolic syndrome, characterized by hepatic lipid accumulation, insulin resistance, and low-grade chronic inflammation. Increasing evidence implicates gut–liver axis disturbances, including dysbiosis-induced intestinal permeability, as key drivers of MASLD progression [24]. Rebamipide’s established human safety profile, validated over 30+ years of clinical use across Asia and Russia, strongly supports its repurposing feasibility for MASH. A 2019 meta-analysis of 15 RCTs confirms mild, self-resolving AEs in ~36% of patients—far fewer than lansoprazole (31.5% vs. 65%)—with no serious events, negligible CYP450 interactions, and Russian guideline endorsement (Rebagit^®^; Level A for H. pylori) [11]. Its high plasma protein binding (98.4–98.6%) and targeted accumulation in stomach, intestines, liver, and kidneys further enhance its favorable pharmacokinetic safety for clinical translation [25]. In this study, proteomic profiling of a MHFD model revealed profound metabolic derangement that closely recapitulates features of advanced MASLD, including hepatic steatosis, mitochondrial dysfunction, oxidative stress, and early fibrogenic activation. Our analysis showed widespread downregulation of core metabolic enzymes, including arginase-1 (urea cycle) and pyruvate kinase PKLR (glycolysis), highlighting a global disruption of energy metabolism. This aligns with prior studies demonstrating MHFD-induced impairment of insulin signaling, glucose utilization, and mitochondrial oxidative capacity [26,27]. GO enrichment further supported this pattern, revealing significant enrichment of carboxylic acid, organic acid, and small-molecule metabolic processes, consistent with nutrient overload and stress-driven catabolism. Enrichment of small-molecule catabolic and amino acid metabolic pathways suggests a compensatory shift toward substrate degradation and detoxification, reflecting hepatic stress adaptation to lipid excess [28]. Notably, molecular function analysis revealed enrichment in catalytic activity categories, yet these were dominated by enzymes whose overall abundance was reduced. This pattern suggests an energy-deficient, stress-adapted hepatic state, consistent with early metabolic inflexibility and a predisposition to chronic metabolic disorders [29,30]. Such proteomic remodeling is well-documented in high-fat diet models and is associated with lipid-handling defects, oxidative stress vulnerability, and impaired xenobiotic metabolism [31,32,33]. These findings align with clinical MASLD, where dysregulated lipid trafficking, mitochondrial injury, and redox imbalance contribute to disease progression and systemic insulin resistance [34,35]. Collectively, these data confirm that MHFD feeding induces broad reprogramming of hepatic metabolism, favoring catabolic over anabolic processes and reflecting a maladaptive attempt to maintain homeostasis under lipid overload. This model successfully reproduces the molecular hallmarks of early MASLD and provides a robust platform for therapeutic intervention studies. Furthermore, specific proteins such as arginase-1 and PKLR emerge as candidate biomarkers of early metabolic stress, while their regulatory pathways may represent promising therapeutic targets for intervention. The integration of GO enrichment analysis proved essential in contextualizing these proteomic shifts, underscoring its utility in uncovering systems-level mechanisms driving metabolic dysfunction [9,36,37].

Rebamipide, a mucosal protectant with well-documented antioxidant and microbiota-modulating properties, has emerged as a promising candidate for metabolic liver disease therapy by restoring gut barrier integrity and dampening inflammatory signaling [15]. In this study, we used proteomics to investigate the hepatic effects of Rebamipide in a MHFD-induced MASLD model, focusing on its prophylactic (Reb T1) or therapeutic (Reb T2) effects. The proteomic landscape revealed that Rebamipide exerts distinct yet complementary effects: prophylactic administration activates protective metabolic pathways to prevent disease onset, while therapeutic treatment drives systemic remodeling of hepatic metabolism to reverse established MASLD pathology. Prophylactic treatment (Reb T1) produced early and targeted metabolic corrections, restoring specific lipid-handling and oxidative stress-related proteins. Notably, the downregulation of fatty acid binding proteins (FABP7) and pyruvate carboxylase, along with the upregulation of prostaglandin reductase 1, suggests that Rebamipide acts early to suppress lipid uptake, improve fatty acid utilization, and modulate inflammatory mediators. These effects align with prior reports demonstrating that Rebamipide improves lipid profiles, reduces hepatic steatosis, and enhances insulin sensitivity in high-fat diet models [38]. GO enrichment supported these findings, with significant modulation of organic acid metabolism, gluconeogenesis, and nucleotide binding pathways, suggesting the initiation of a therapeutic metabolic reprogramming process [39,40]. The enrichment of unique enzyme activities, such as ceramide 1-phosphate transfer and flavonol 3-sulfotransferase, further implies early engagement of specific lipid and signaling networks critical for hepatocyte protection [41,42,43]. These molecular changes support the hypothesis that prophylactic Rebamipide mobilizes a rapid, targeted protective response, providing metabolic stability and resistance to MHFD-induced hepatic injury. Therapeutic intervention (Reb T2) demonstrated broader, systemic metabolic reprogramming. Proteomic analysis revealed upregulation of mitochondrial and energy metabolism proteins, including actinin alpha-4, adenylate kinase 2, and phosphoglycerate mutase, consistent with improved mitochondrial adaptation and enhanced energy production. Conversely, tubulin alpha chains were downregulated, suggesting normalization of cytoskeletal remodeling and improved insulin signaling, a key defect in MHFD-induced metabolic stress [44,45]. GO enrichment indicated a shift toward fatty acid β-oxidation, monocarboxylic acid catabolism, and organic acid catabolism, suggesting that Rebamipide promotes efficient lipid degradation and detoxification during sustained dietary stress [41,42,43]. Together, these changes indicate that therapeutic Rebamipide treatment moves beyond acute correction to establish a stable, adaptive metabolic phenotype that enhances mitochondrial resilience, restores glucose and lipid homeostasis, and reduces lipotoxic stress. The dual-phase action of Rebamipide is supported by unsupervised clustering and volcano plot analyses, which revealed early modulation of stress response proteins, followed by widespread remodeling of mitochondrial and energy networks during extended therapy. This phased progression suggests that Rebamipide initially mobilizes rapid molecular defenses, followed by comprehensive reprogramming of metabolic pathways to stabilize energy balance and inflammation control. These findings align with prior evidence showing Rebamipide’s capacity to modulate immune function, enhancing regulatory T-cell and M2 macrophage activity while suppressing proinflammatory Th17 and M1 macrophage polarization, as well as its inhibition of adipocyte differentiation [38,42,46]. Such immunomodulatory properties likely complement its metabolic actions, helping to suppress diet-induced inflammation while restoring hepatocellular resilience. Collectively, our findings demonstrate that Rebamipide exerts both prophylactic and therapeutic effects in MASLD. Prophylactic administration prevents early oxidative stress and preserves lipid homeostasis, whereas therapeutic treatment reverses established metabolic dysfunction by restoring mitochondrial integrity, improving insulin sensitivity, and re-establishing hepatic metabolic balance.

**Figure 13:**
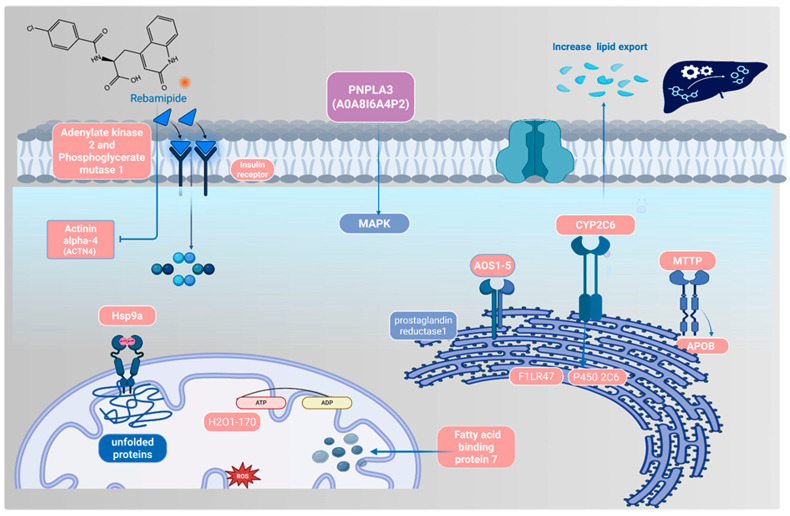
Proposed mechanistic model of Rebamipide’s therapeutic and preventive actions in MASLD. Schematic representation illustrating Rebamipide’s multi-layered mode of action in MHFD-induced liver injury, including sequential activation of xenobiotic detoxification, mitochondrial defense and protein import pathways, and lipid mobilization and export mechanisms. This coordinated metabolic reprogramming underlies Rebamipide’s dual role in preventing MASLD onset and reversing established steatosis, highlighting its potential as a repurposable therapeutic for metabolic liver disease.

Comparative proteomic profiling across all three experimental contrasts (control vs. MHFD, MHFD vs. Reb T1, and MHFD vs. Reb T2) identified nine consistently dysregulated proteins (D4A1W8, F1M953, A0A8I5ZR52, P48721, F1LR47, P05178, Q5EB99, A0A8I6A4P2, and A0A1B0GWL1) that represent central molecular nodes of MHFD-induced hepatic dysfunction. These included mitochondrial stress-70 chaperones, microsomal triglyceride transfer protein (MTTP), and multiple cytochrome P450 monooxygenases, reflecting key roles in mitochondrial proteostasis, lipid trafficking, and detoxification. All were strongly downregulated in MHFD-fed rats, indicating widespread impairment of lipid handling and cellular stress responses. Both prophylactic and therapeutic Rebamipide treatment reversed these changes, with therapeutic exposure achieving robust restoration and even supraphysiological upregulation of MTTP and mitochondrial stress proteins, suggesting that Rebamipide drives a metabolic reprogramming effect that enhances triglyceride export, mitochondrial import machinery, and detoxification capacity. The upregulation of cytochrome P450 isoforms also points to improved xenobiotic clearance, which is critically needed in oxidative stress–prone metabolic states [12,47,48,49,50]. These findings extend Rebamipide’s known antioxidant and anti-inflammatory effects by showing direct proteomic correction of lipid and mitochondrial pathways. Data mining using the Rat Genome Database (RGD) mapped these proteins to three central genes, Mttp, Cyp2c6, and Hspa9, with varying levels of evidence linking them to MASLD pathogenesis. Mttp, encoding MTTP, is essential for the assembly and secretion of ApoB-containing lipoproteins, including very-low-density lipoprotein (VLDL) in the liver and chylomicrons in the intestine [51]. Mutations in the human ortholog MTTP cause abetalipoproteinemia, a disorder characterized by severe fat malabsorption and hepatic steatosis [52,53], while animal models confirm its role in lipid metabolism and steatosis development [54,55]. Cyp2c6 encodes a cytochrome P450 enzyme involved in fatty acid metabolism, drug processing, and xenobiotic clearance, with relevance to dyslipidemia, hepatocellular carcinoma, and type 2 diabetes [38,56,57] (Figure 13). Though not yet directly linked to MASLD, CYP2C19—the human ortholog—is a well-studied pharmacogenomic variant affecting drug metabolism and lipid regulation, warranting further investigation in metabolic liver disease. Hspa9, encoding a mitochondrial heat-shock protein, regulates protein import, folding, and oxidative stress response and is linked to mitochondrial dysfunction syndromes such as sideroblastic anemia [58] and neurodevelopmental disorders [59]. Its role in preserving mitochondrial integrity supports it as a secondary MASLD candidate gene [60]. Network and enrichment analyses positioned these genes at the intersection of lipid export, cytochrome P450-mediated detoxification, mitochondrial stress signaling, and MAPK-driven inflammatory regulation [52,53,54]. GO and Reactome analyses highlighted disruptions in triglyceride transport, ceramide metabolism, PUFA processing, xenobiotic oxidation, and protein–lipid complex assembly [55,61], all corrected with Rebamipide treatment. Pathway-level interrogation via KEGG and Reactome reinforced this, showing dysregulation in fat digestion and absorption, steroid biosynthesis, lipoprotein remodeling, and cytochrome P450 oxidation networks, placing Mttp, Cyp2c6, and Hspa9 at key metabolic choke points [1,38,61,62].Disease ontology mapping connected these molecular signatures to metabolic syndrome, hyperlipoproteinemia type II, hyperinsulinism, and hepatocellular carcinoma, demonstrating the clinical relevance of this MHFD model. Collectively, these results show that MHFD triggers a profound metabolic collapse involving mitochondrial proteostasis, detoxification, and lipid trafficking, while Rebamipide corrects these pathways through coordinated, multi-targeted proteomic remodeling. The nine identified proteins form a compact biomarker panel for MASLD progression and treatment response, and Mttp, Cyp2c6, and Hspa9 emerge as mechanistically important targets for diagnostic and therapeutic exploration. Together, these findings emphasize that Rebamipide’s therapeutic benefits extend well beyond its anti-inflammatory properties, encompassing metabolic reprogramming, redox defense, and xenobiotic processing, positioning it as a promising repurposable treatment for diet-induced metabolic liver disease. To validate our proteomic findings, we performed a detailed histopathological assessment using H&E and PAS staining, which provided morphological confirmation of MHFD-induced hepatic injury and the restorative effects of Rebamipide treatment. In the MHFD-induced model group, liver sections showed extensive steatosis, with hepatocytes displaying cytoplasmic lipid droplets, altered polygonal architecture, and cytoplasmic rarefaction, consistent with lipid accumulation and metabolic stress as reported in other high-fat-diet-induced MASLD models [63]. Activation of Ito (hepatic stellate) cells containing lipid vacuoles was prominent in the space of Disse, indicative of early fibrogenic activity, while portal tracts exhibited mononuclear inflammatory infiltrates, reflecting the low-grade hepatic inflammation typical of MASLD progression [59]. PAS staining further revealed marked glycogen depletion, highlighting impaired glucose handling and disrupted hepatic energy homeostasis. In contrast, Rebamipide-treated groups demonstrated significant morphological improvement, aligning with its known antioxidant, anti-inflammatory, and mucosal-protective properties [38,60]. The therapeutic treatment group (Reb T2) displayed a near-complete restoration of hepatic architecture, with reduced lipid accumulation, fewer activated stellate cells, and normalization of hepatocyte structure, while PAS staining revealed robust glycogen reaccumulation, signifying improved metabolic regulation. The prophylactic group (Reb T1) also showed reduced lipid burden and partial structural recovery, suggesting early protective effects against MHFD-induced injury. These findings corroborate recent work demonstrating Rebamipide’s ability to attenuate lipid accumulation, improve insulin sensitivity, and limit diet-induced hepatic inflammation [64,65], now extended here with direct histological evidence. Collectively, these histopathological results confirm that Rebamipide exerts both preventive and therapeutic effects in MASLD, complementing proteomic findings that revealed improvements in mitochondrial function, lipid trafficking, and oxidative stress defense. The reversal of steatosis, reduction in stellate cell activation, and restoration of glycogen content provide strong morphological evidence that Rebamipide can correct both metabolic and structural hallmarks of MASLD, supporting its potential as a repurposable therapy for metabolic liver disease.

The mucosal protective characteristics of Rebamipide have been studied extensively and it has been shown to increase production of prostaglandins as well as to scavenge reactive oxygen species, and to stimulate the secretion of mucus and mucin in the gastrointestinal tract [11,12,66]. Research indicates that it increases intestinal barrier integrity by promoting the secretion of MUC2 from goblet cells via the Akt signaling pathway, decreases NAID-induced increases in intestinal permeability, promotes the integrity of tight junctions, and reduces oxidative and inflammatory injury [15,67]. It was shown to reduce intestinal permeability, improve the integrity of inter-cellular tight junctions, and decrease the levels of intestinal MDA and MPO activity. At the mitochondrial level, it was shown to increase the activities of SDH and ATPase, increase levels of NADH, and decrease mitochondrial swelling [68]. In addition, new evidence suggests that Rebamipide can modulate the composition of the microbiota in the small intestine in the setting of drug-induced enteropathy [15]. Beyond the stomach, Rebamipide has protective effects in the small intestine by reducing inflammation and preserving barrier function. It also positively affects gut microbiota and increases mucosal blood flow, thereby providing comprehensive gastrointestinal protection. thus, reports support using Rebamipide as a safe and effective option for preventing and treating aspirin-associated gastroduodenopathy [69]. While our study focuses on the proteomic remodeling of the liver along with histopathological changes due to experimental MHFD-induced MASLD, we did not assess gut permeability or microbiome composition directly, and as such, we acknowledge these as limitations of our work and priorities for future mechanistic investigation. A limitation of the present study is that only male Wistar rats were used. Sex-related differences in MASLD pathogenesis have been widely reported, with estrogen-mediated protective mechanisms reducing hepatic lipid accumulation and inflammatory responses in females. Consequently, disease progression and therapeutic responses may differ between sexes. Future investigations should therefore include female animal models and clinical cohorts to better characterize potential sex-specific effects of Rebamipide in metabolic liver disease.

Overall, this multi-level proteomic and histological study demonstrates that Rebamipide exerts both prophylactic and therapeutic effects against MHFD-induced hepatic dysfunction, acting through complementary but distinct mechanisms. Prophylactic administration preserved mitochondrial integrity, oxidative stress defenses, and lipid homeostasis, effectively preventing early metabolic injury, while therapeutic treatment reversed established steatosis by restoring lipid trafficking through Mttp and reprogramming detoxification and stress-response pathways, including Cyp2c6- and Hspa9-mediated functions. Enrichment analyses further revealed Rebamipide’s broad influence on lipid handling, xenobiotic metabolism, bile acid synthesis, and MAPK-mediated inflammatory regulation, consistent with its known anti-inflammatory activity via suppression of cytokines such as IL-1β and TNF-α [38,60]. These findings highlight Rebamipide as a promising candidate for repurposing in MASLD management, with the potential to both prevent disease onset and remodel metabolic networks in established pathology.

## 4. Materials and Methods

### 4.1. Reagents and Drugs

Rebamipide was purchased from Otsuka Pharmaceutical Co. (Tokyo, Japan). The high-fat liquid diet (MHFD; Lieber-DeCarli formulation) was prepared using base components sourced from El Gomhoreya Chemicals Co. (Cairo, Egypt). For histological processing, tissue fixation was carried out using 38% formaldehyde solution (El Gomhoreya Chemicals Co., Cairo, Egypt). Physiological saline (0.9% NaCl) was supplied in sterile 500 mL solutions (El Nile Pharmaceutical Co., Cairo, Egypt).

### 4.2. Experimental Design and Animal Groups

**Figure 14:**
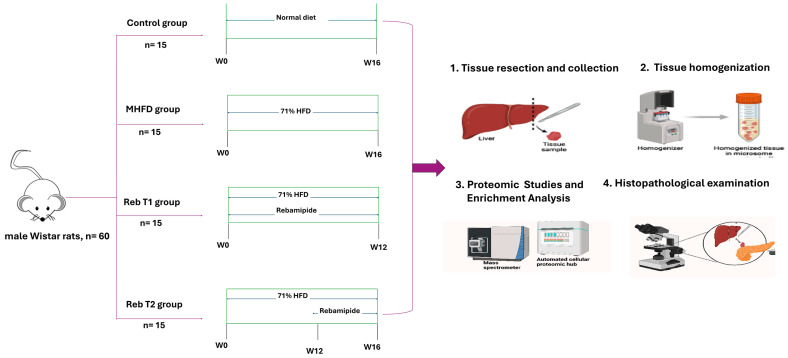
Schematic representation of treatment protocols and animal groups or Rebamipide administration in a 4-month NASH model.

All experimental procedures were conducted in compliance with the Animals (Scientific Procedures) Act of 1986 and related ethical guidelines. The study protocol was approved by the Research Ethics Committee for Experimental and Clinical Studies, Faculty of Science, Port Said University (Approval code: PSU.Sci22, 20 November 2023). A total of 60 healthy, immunocompetent male Wistar rats (8 weeks old, 200 ± 30 g) with no prior experimental procedures were acclimatized under standard conditions (22 ± 2 °C, 50–60% humidity, 12 h light/dark cycle) with free access to food and water, with the individual animal as the experimental unit. To minimize potential confounders, all animals were housed under identical environmental conditions, and treatments, sample collections, and measurements were performed in a consistent sequence across all groups during the same time frame each day. Cage positions were rotated weekly to avoid location-related effects. Group allocation and treatment administration were performed by personnel who were not involved in the subsequent outcome assessments. The sample size was determined a priori using G*Power software version 3.1.9.2 (2017), following standard assumptions for comparing multiple independent means with ANOVA and post hoc tests (α = 0.05, two-tailed, 80% power, effect size f = 0.4 based on pilot MHFD data for ALT, triglycerides, and glucose). This yielded 10 animals per group, reflecting a balance between statistical rigor and ethical considerations in accordance with the 3Rs principle. Accordingly, rats were randomly assigned using simple randomization to four predefined groups (n = 10 per group), Figure 14:

1. Control group (fed a standard chow diet).
2. MHFD-model group that was fed a high-fat liquid diet The MHFD model group received a high-fat liquid diet (71% energy from fat: corn oil (48.5 g/kg), olive oil (28.4 g/kg), safflower oil (2.7 g/kg); 11% carbohydrates: dextrin maltose (25.6 g/kg); 18% protein: casein (41.4 g/kg), L-cystine (0.5 g/kg), dl-methionine (0.3 g/kg)) ad libitum for 16 weeks [70].
3. Prophylactic group (Reb T1), receiving Rebamipide (100 mg/kg/day, p.o., dissolved in 0.9% saline) concomitantly with the high-fat diet starting from week 0.
4. Therapeutic group (Reb T2), fed a high-fat diet for 12 weeks to induce NASH, followed by Rebamipide treatment (100 mg/kg/day, p.o) during the final 4 weeks [71].

The experimental design was based on prior studies and our established MHFD model, in which hepatic steatosis develops within 4 weeks, early NASH with inflammation and hepatocyte ballooning appears by 8–12 weeks [24]. NASH progression was confirmed at week 12 using predefined diagnostic thresholds (ALT > 80 U/L, triglycerides > 150 mg/dL, fasting glucose > 200 mg/dL, low-density lipoprotein (LDL)/high-density lipoprotein (HDL) ratio > 2.5), and non-responders failing to meet these criteria were excluded. Serial blood samples were collected from the tail vein at weeks 0 (baseline), 4, 8, 12, and 16 to monitor serum biochemical markers, including ALT, triglycerides, fasting glucose, LDL, HDL, LDL/HDL ratio, and body weight (Figure S1). For proteomic analysis, liver tissues from three animals per experimental group (*n* = 3 biological replicates) were selected and subjected to LC–MS/MS analysis. The remaining animals in each group were allocated for histopathological and morphometric assessments to validate the molecular findings. The Rebamipide dose (100 mg/kg/day, p.o.) was selected based on previous studies demonstrating hepatoprotective and anti-inflammatory effects in rodent models of metabolic disease. Doses in the range of 30–100 mg/kg/day have been reported to be effective and safe in high-fat diet models. Based on body surface area normalization, this dose corresponds to a human equivalent dose of approximately 16 mg/kg, which is within the pharmacological exposure range of clinically used Rebamipide.

### 4.3. Sample Processing

Animals were humanely euthanized at the end of the experimental period by decapitation under deep anesthesia induced by a ketamine/xylazine combination, following standard protocol [72]. The procedure was performed in accordance with the institutional ethical guidelines and the AVMA Guidelines for the Euthanasia of Animals (2020) to ensure minimal pain and distress. The whole liver was immediately excised, dissected, and rinsed thoroughly with ice-cold 0.9% saline to remove residual blood before processing. Liver tissues were homogenized on ice in lysis buffer (8 M urea, 50 mM Tris-HCl, pH 8.5, supplemented with protease inhibitor cocktail) using an ultrasonic homogenizer. The homogenates were centrifuged at 10,000× *g* for 30 min at 4 °C, and protein concentrations in the supernatants were quantified using the bicinchoninic acid assay (Thermo Fisher Scientific, Waltham, MA, USA). For proteomic analysis, aliquots containing 100 μg total protein were denatured in 8 M urea and 50 mM Tris-HCl (pH 8.5), reduced with 10 mM dithiothreitol at 37 °C for 1 h, and alkylated with 20 mM iodoacetamide in the dark at room temperature for 30 min. Proteins were digested overnight at 37 °C with trypsin (1:50 enzyme-to-protein ratio, Promega, Madison, WI, USA). The digestion reaction was quenched with 1% formic acid, and peptides were desalted using C18 spin columns (Thermo Fisher Scientific, USA) and vacuum-dried. A final volume of 10 μL peptide solution containing 1 μg peptides was prepared for injection, and all samples were analyzed in technical duplicates by LC-MS/MS [73,74].

### 4.4. Proteomic Data Analysis

Proteomic analysis was performed on a NanoLC system comprising an Eksigent nanoLC 400 (Eksigent Technologies LLC, Dublin, CA, USA) autosampler coupled to an Eksigent Ekspert nanoLC425 pump (Eksigent Technologies LLC) and interfaced with a Sciex TripleTOF™ 5600+ LC-QTOF mass spectrometer (Sciex, Framingham, MA, USA) operating in positive ion acquisition mode. Samples were analyzed using a trap-and-elute workflow with a total run time of 120 min per sample. Peptides were first loaded onto a CHROMXP C18CL trapping cartridge (5 μm, 10 × 0.5 mm) at 10 μL/min in mobile phase A (0.1% formic acid in Milli-Q water) for 3 min, followed by separation on an analytical column (CHROMXP C18CL, 3 μm, 120 Å, 150 × 0.3 mm) at 10 μL/min. Mobile phase B consisted of 0.1% formic acid in acetonitrile. A needle wash with 10% isopropanol was performed twice between injections to minimize sample carryover. Mass spectrometry was conducted in information-dependent acquisition (IDA) mode. Survey TOF-MS scans (MS1) were acquired at 400–1250 m/z, followed by MS/MS (MS2) product ion scans at 170–1500 m/z for the top 40 most abundant precursor ions per cycle, with a cycle time of 1.5 s. An ion intensity threshold of 150 cps was applied for precursor selection. External calibration was performed prior to sample acquisition using the Sciex tuning solution (P/N 4457953) to ensure mass accuracy. All sample runs were completed within 120 min, and each sample was analyzed in technical duplicate [73,74,75].

### 4.5. Processing and Protein Identification

Raw MS data were acquired using Analyst^®^ TF software (v1.7.1, Sciex) and processed with ProteinPilot™ software (v5.0.1.0, build 4895) utilizing the Paragon™ Algorithm (v5.0.1.0, build 4874) for protein identification. Spectra were searched against the UniProt Rattus norvegicus reference proteome (Swiss-Prot and TrEMBL; ~94,580 protein entries). Search parameters specified trypsin digestion, allowing for up to two missed cleavages, with carbamidomethylation of cysteine set as a fixed modification and oxidation of methionine as a variable modification. Peptide and protein identifications were filtered at a 1% global false discovery rate (FDR) using a decoy database search strategy. Proteins identified with ≥1 unique peptide and meeting the FDR threshold were retained for downstream analysis. Relative protein quantification was performed using label-free quantitation (LFQ) based on normalized MS1 ion intensities [75]. After database searching against the *Rattus norvegicus* UniProt reference proteome and filtering, proteins were subjected to preprocessing and missing-value filtering prior to quantitative analysis. The total number of quantified proteins retained for downstream analysis was 3569 proteins for the Control vs. MHFD comparison, 3365 proteins for the MHFD vs. Reb T1 comparison, and 4335 proteins for the MHFD vs. Reb T2 comparison, representing substantial proteome coverage for label-free LC-MS/MS analysis of rat liver tissue

### 4.6. Data Preprocessing

Proteomic intensity data were derived from peptide peak areas mapped to their corresponding parent protein accessions. For each sample, peptide intensities were summed to obtain total protein intensities. To minimize technical variation across runs, data were normalized using probabilistic quotient normalization (PQN) with a designated reference sample. Proteins with missing values in ≥2 samples or with >50% missing values per experimental group were excluded from downstream analyses. Remaining missing values were imputed using median-based random imputation within a ±1% range of the median intensity to preserve data integrity. Finally, data were auto-scaled (mean-centered and divided by standard deviation) to optimize visualization, clustering, and multivariate statistical interpretation.

### 4.7. Differential Protein Identification

To characterize proteomic alterations associated with MASLD and evaluate the impact of Rebamipide therapy, multivariate and univariate statistical analyses were performed. Principal Component Analysis (PCA) and Partial Least Squares Discriminant Analysis (PLS-DA) were conducted using MetaboAnalyst (v5.0) to visualize global proteomic variation and assess group separation. Prior to analysis, data were log-transformed and Pareto-scaled to reduce the influence of large-scale variance while preserving data structure. Proteins contributing most to group discrimination were identified using variable importance in projection (VIP) scores derived from the PLS-DA model, with a threshold of VIP > 1.0. In parallel, univariate analysis was performed using Student’s *t*-test, applying a significance level of *p* < 0.05. Proteins were classified as differentially expressed if they met both statistical criteria (VIP > 1.0 and *p* < 0.05).

### 4.8. Gene Ontology and Pathway Enrichment Analysis

Gene Ontology (GO) and pathway enrichment analyses were performed using g:Profiler [76]. GO terms were explored across three categories: biological process (BP), molecular function (MF), and cellular component (CC), while pathway-level enrichment was conducted using both Kyoto Encyclopedia of Genes and Genomes (KEGG) and Reactome databases. *p*-values were adjusted for multiple testing using the Benjamini–Hochberg false discovery rate (FDR) correction, with statistical significance set at adjusted *p* < 0.05. For each ontology and pathway term, gene counts and adjusted *p*-values were extracted and ranked according to significance. Highly enriched terms were prioritized for biological interpretation and further downstream exploration.

### 4.9. Network Analysis

Gene interaction and functional association networks were constructed using GeneMANIA with the *Rattus norvegicus* database selected as the reference species [24]. The analysis integrated multiple biological evidence types, including physical interactions, co-expression, genetic interactions, pathway associations, and protein domain similarity, to predict functional relationships between the input genes. Pathway enrichment and functional category predictions were performed within GeneMANIA to identify key biological processes, molecular functions, and signaling pathways relevant to the provided gene set. Enriched network modules were further examined to highlight genes of high connectivity and potential regulatory importance in MASLD pathogenesis and Rebamipide response.

### 4.10. Data Mining from Rat Genome Database (RGD)

To investigate gene–disease associations and extract relevant functional annotations, the Rat Genome Database (https://rgd.mcw.edu/) was used as a comprehensive resource for rat genetics, genomics, and translational research. RGD integrates curated data on rat genes, proteins, phenotypes, pathways, and their orthologous relationships to human diseases, making it a valuable platform for comparative studies. Genes of interest identified from proteomic analyses were queried in RGD to retrieve curated disease annotations, phenotypic data, and human ortholog information, with a focus on experimental evidence curated from databases such as ClinVar and OMIM. Search prioritized orthology-based disease associations, particularly those related to MASLD (formerly MAFLD), metabolic dysfunction, and related systemic phenotypes. Annotation data were critically reviewed for relevance, and disease models with pathophysiological parallels to MASLD and MHFD-induced phenotypes, including neurodevelopmental disorders, anemia, and mitochondrial dysfunction, were examined to strengthen translational interpretation.

### 4.11. Histopathological Examination

Liver tissues from all experimental groups were collected immediately after sacrifice and fixed in 4% paraformaldehyde (PFA) for at least 24 h to preserve tissue architecture. Fixed samples were thoroughly washed, dehydrated in ascending ethanol concentrations (70%, 80%, 90%, 95%, and 100%), cleared in xylene, and embedded in paraffin wax. Serial 3 μm thick paraffin sections were cut using a rotary microtome (Leica RM2125, Wetzlar, Germany) and mounted on glass slides for histological evaluation. Two staining protocols were applied: hematoxylin and eosin (H&E) staining was used to examine general hepatic morphology, including hepatocyte structure, fat deposition, inflammatory infiltrates, and stellate (Ito) cell activation. To morphometrically assess the degree of hepatic steatosis, the activation status of hepatic stellate (Ito) cells was evaluated in H&E-stained liver sections. Quiescent Ito cells were identified as small, flattened, stellate-shaped cells located in the perisinusoidal space. Activated Ito cells were recognized by their enlarged, polygonal or spindle-shaped, myofibroblast-like morphology. From each experimental group, randomly selected H&E-stained fields were captured at ×10 magnification from five rats. In each captured image, the total number of Ito cells and the number of morphologically activated Ito cells were manually counted using ImageJ (version 1.32j, National Institutes of Health, Bethesda, MD, USA). The percentage of activated Ito cells was calculated as follows: percentage of activated Ito cells = (number of activated Ito cells/Total number of Ito cells) × 100. The calculated percentages were statistically compared among the studied groups. Periodic acid–Schiff (PAS) staining was employed to detect glycogen and mucopolysaccharide deposition as indicators of hepatocellular injury and metabolic dysfunction. For each animal, at least four representative fields were analyzed per slide, with five animals per group, yielding a total of 20 fields per experimental group. Histological examinations were performed using a Carl Zeiss light microscope (Zeiss, Berlin, Germany) at 20×, 40×, and 100× magnification. Tissue evaluation focused on key parameters, including hepatocyte vacuolation, steatosis severity, inflammatory cell infiltration, Ito cell activation, and glycogen storage levels, allowing for a comprehensive assessment of MHFD-induced pathology and the effects of Rebamipide treatment.

### 4.12. Statistical Analysis

Prior to differential expression analysis, the distribution of proteomic intensity values was evaluated using the Shapiro–Wilk normality test. As several datasets deviated from normal distribution, statistical comparisons between groups were performed using the non-parametric Mann–Whitney U test. This approach was also considered appropriate given the limited number of biological replicates used for proteomic analysis (*n* = 3 per group). Proteomics data were first normalized to correct for systematic variation, filtered to retain only features detected in at least 50% of all samples, and subjected to random median imputation to address missing values, ensuring high-quality input for downstream analysis. Differential protein expression was assessed through pairwise comparisons among the four experimental groups (control, MHFD, Reb T1, and Reb T2) using the non-parametric Mann–Whitney (Wilcoxon rank-sum) test, chosen for its robustness and minimal assumptions regarding data distribution, which is well-suited for proteomic datasets. Proteins were classified as significantly differentially expressed if they satisfied both statistical significance (two-sided *p* ≤ 0.05) and biological relevance (|log_2_ fold change| ≥ 1) thresholds. Results were visualized using volcano plots, which simultaneously display both fold-change magnitude and statistical confidence, enabling rapid identification of key proteins associated with MHFD-induced pathology and Rebamipide treatment response. Histological morphometric data were analyzed statistically for the percentage of activated Ito cells using GraphPad Prism version 8.0.0(GraphPad Software, San Diego, CA, USA). Data were analyzed using two-way analysis of variance (ANOVA) to evaluate differences among the studied groups, followed by Tukey’s post hoc multiple comparison test when a significant overall effect was detected. Results are presented as mean ± standard error of the mean (SEM). Differences were considered statistically significant at *p* < 0.05 and highly significant (**) at *p* < 0.01.

## 5. Conclusions

This study provides comprehensive mechanistic insight into the hepatoprotective potential of Rebamipide in MASLD, using an integrative approach that combined high-resolution proteomics, functional enrichment, network analysis, and histological validation. Our findings reveal that Rebamipide acts through a dual protective and therapeutic mechanism: prophylactic administration prevents metabolic derangements by rapidly engaging detoxification and stress-response pathways, while therapeutic intervention restores lipid trafficking, mitochondrial function, and lipoprotein assembly, thereby reversing advanced hepatic steatosis and structural injury. The identification of nine consistently dysregulated proteins and three core regulatory genes (Mttp, Cyp2c6, and Hspa9) highlights central molecular nodes of MASLD progression and underscores Rebamipide’s ability to reprogram lipid metabolism, enhance antioxidant defense, and modulate inflammatory signaling. These discoveries not only clarify Rebamipide’s multifaceted mechanism of action but also provide candidate biomarkers for disease monitoring and pharmacological response evaluation. By integrating molecular profiling with histopathological evidence, this work positions Rebamipide as a promising repurposable drug for metabolic liver diseases. Future studies should validate these proteomic signatures in human cohorts, examine their role in gut–liver axis regulation, explore synergy with emerging MASLD therapies, and include female models to address post-menopausal MASH relevance. Overall, this study advances our understanding of MASLD pathogenesis, establishes a molecular framework for precision therapeutic development, and paves the way for translating Rebamipide into a clinically relevant strategy for preventing and reversing diet-induced metabolic liver injury.

## Data Availability

The original contributions presented in this study are included in the article/Supplementary Material. Further inquiries can be directed to the corresponding author.

## Supplementary Materials

The following supporting information can be downloaded at: https://www.mdpi.com/article/10.3390/ph19040559/s1, Figure S1: Assessment of NASH-associated metabolic and biochemical markers in experimental groups compared to the control group. (**A**) Body weight, (**B**) fasting serum glucose, (**C**) alanine aminotransferase (ALT), (**D**) triglycerides (TG), (**E**) low-density lipoprotein (LDL), and (**F**) high density lipoprotein (HDL) levels. Data are presented as mean ± SD; Figure S2: Quality control and data pre-processing of proteomic datasets at both sample and protein levels, shown before and after normalization and filtering. Panels represent (**A**) Control vs. MHFD, (**B**) MHFD vs Rebamipide prophylactic group (Reb T1), and (**C**) MHFD vs Rebamipide therapeutic group (Reb T2); Figure S3: Photomicrograph of hematoxylin and eosin (H&E) stained rat liver sections from the MHFD group. Sections show largely preserved hepatic architecture with focal mononuclear cellular infiltration observed in the portal tracts of some lobules.
