# Supplementary material for: Rebamipide Reprograms Hepatic Networks to Prevent and Reverse Metabolic-Dysfunction-Associated Steatotic Liver Disease: Multi-Omics Insights and Histological Validation
Source: Pharmaceuticals (Basel). 2026 Mar 31;19(4):559. doi: 10.3390/ph19040559 (PMC13119257; doi:10.3390/ph19040559)
Supplement: Supplementary file 1 [file pharmaceuticals-19-00559-s001.zip › pharmaceuticals-4140396-supplementary.pdf]

Supplementary Information

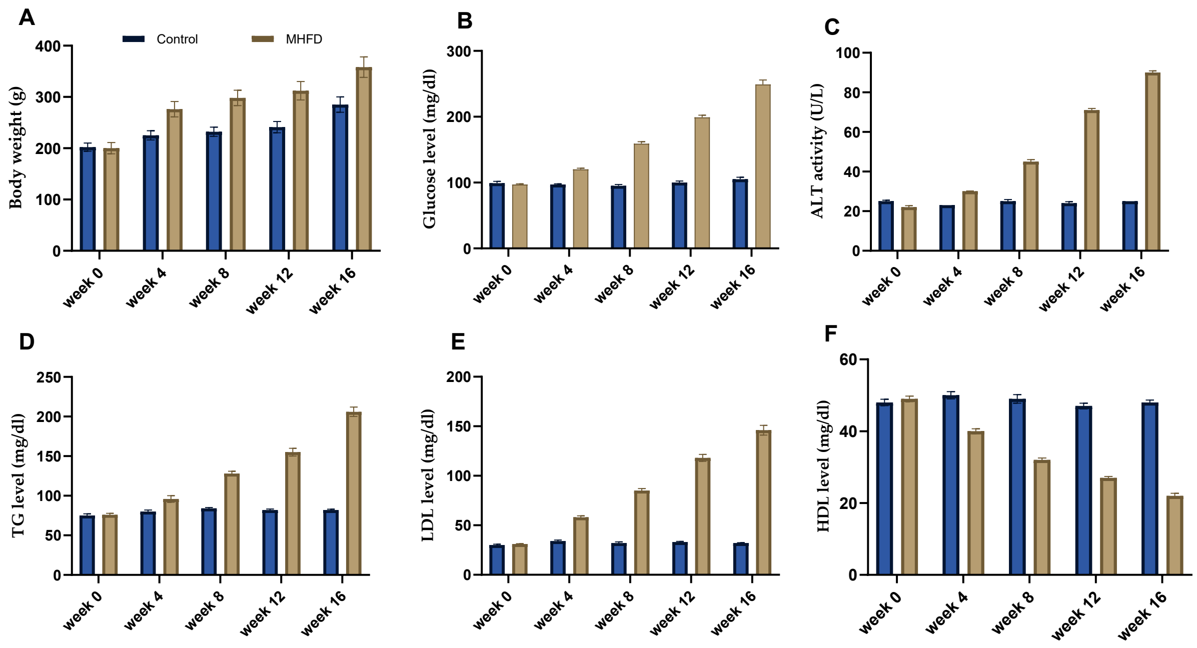

Figure S1: Assessment of NASH-associated metabolic and biochemical markers in experimental groups compared to the control group. (**A**) Body weight, (**B**) fasting serum glucose, (**C**) alanine aminotransferase (ALT), (**D**) triglycerides (TG), (**E**) low-density lipoprotein (LDL), and (**F**) high-density lipoprotein (HDL) levels. Data are presented as mean ± SD.

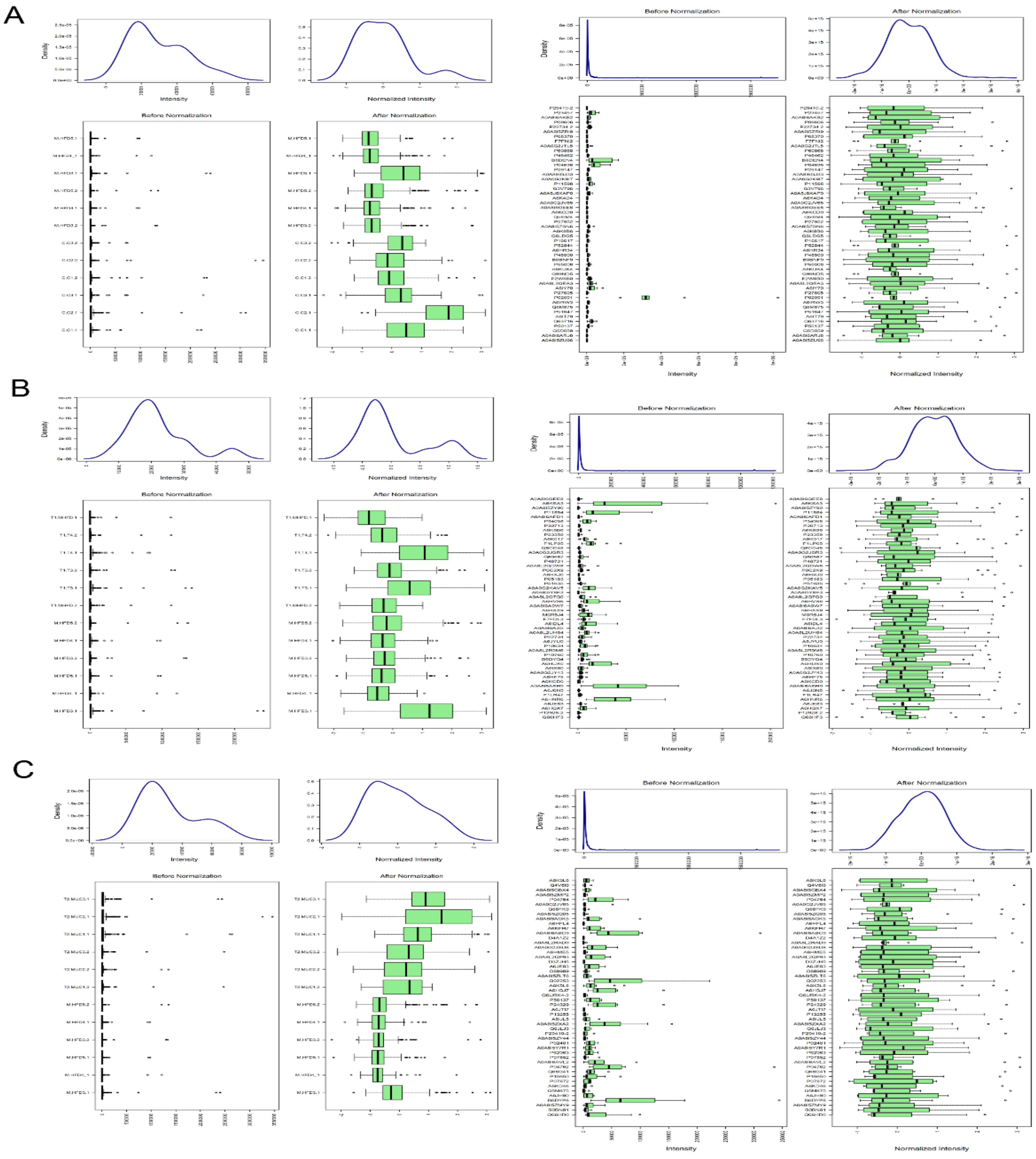

Figure S2: Quality control and data pre-processing of proteomic datasets at both sample and protein levels, shown before and after normalization and filtering. Panels represent (**A**) Control *vs* MHFD, (**B**) MHFD *vs* Rebamipide prophylactic group (Reb T1), and (**C**) MHFD *vs* Rebamipide therapeutic group (Reb T2)

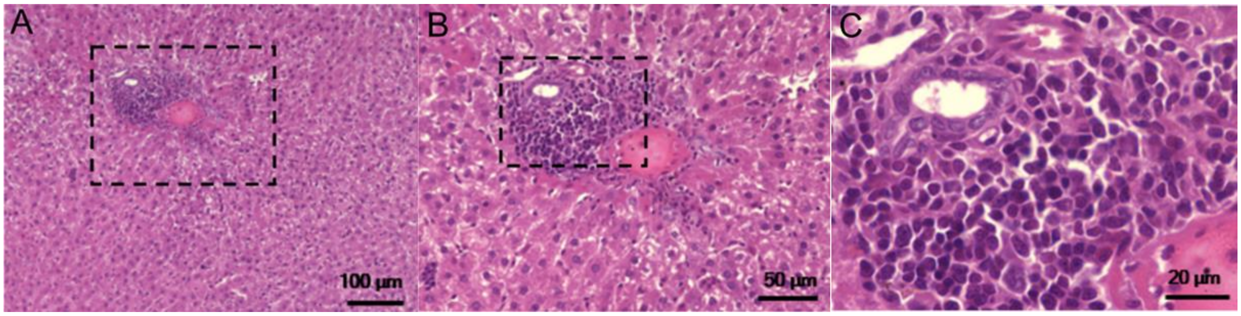

Figure S3: Photomicrograph of hematoxylin and eosin (H&E) stained rat liver sections from the MHFD group. Sections show largely preserved hepatic architecture with focal mononuclear cellular infiltration observed in the portal tracts of some lobules.
